# Monitoring concentration and lipid signature of plasma extracellular vesicles from HR^+^ metastatic breast cancer patients under CDK4/6 inhibitors treatment

**DOI:** 10.1002/jex2.70013

**Published:** 2024-12-17

**Authors:** Mathilde Richard, Rosalie Moreau, Mikaël Croyal, Laurent Mathiot, Jean‐Sébastien Frénel, Mario Campone, Aurélien Dupont, Julie Gavard, Gwennan André‐Grégoire, Laëtitia Guével

**Affiliations:** ^1^ Team SOAP, Centre de Recherche en Cancérologie et Immunologie Intégrée Nantes‐Angers (CRCI^2^NA), Inserm CNRS, Nantes Université Nantes France; ^2^ Équipe Labellisée Ligue Contre le Cancer Paris France; ^3^ Nantes Université, CHU Nantes, CNRS, INSERM Nantes France; ^4^ Nantes Université, CHU Nantes, Inserm, CNRS, SFR Santé, Inserm UMS 016, CNRS UMS 3556 Nantes France; ^5^ CRNH‐Ouest Mass Spectrometry Core Facility Nantes France; ^6^ Institut de Cancérologie de l'Ouest (ICO), Site Rene Gauducheau Saint Herblain France; ^7^ SFR UMS CNRS 3480, INSERM 018, Biosit biologie, santé, innovation technologique Rennes France

**Keywords:** cancer, CDK4/6 inhibitors, circulating biomarkers, extracellular vesicles, sphingolipids

## Abstract

Extracellular vesicles (EVs) are cell‐derived small membrane structures that transport various molecules. They have emerged as potential circulating biomarkers for monitoring responses to cancer therapies. This study aimed to comprehensively characterize plasma‐carried EVs in hormone receptor‐positive (HR^+^) metastatic breast cancer (MBC) patients treated with first‐line CDK4/6 inhibitors (iCDK4/6) combined with endocrine therapy. MBC patients were classified into three groups based on their response to therapy: resistant, intermediate or sensitive. In a prospective cohort, we monitored the concentration of circulating EVs, analyzed their lipid signature and correlated these factors with treatment response. To facilitate the translation of EV research to clinical practice, we established a three‐step procedure: (1) EVs were isolated from plasma using semi‐automatized size exclusion chromatography (SEC); (2) EV concentration, termed vesiclemia, was determined by drop counting via interferometric light microscopy (ILM); and (3) EV lipid composition was analyzed by mass spectrometry. ILM‐based vesiclemia values were highly fluctuating upon iCDK4/6 treatment, while early increase associated with accelerated progression. Of note, vesiclemia remained a steady parameter over a 1‐year period in age‐matched healthy women. Additionally, analysis of the EV cargo unveiled a distinct sphingolipid profile, characterized by increased levels of ceramides and sphingomyelins in resistant patients within the first 2 months of treatment. Based on 16 sphingolipid species, sensitive and resistant patients were correctly classified with an overall accuracy of 82%. This specific sphingolipid pattern was exclusively discernible within EVs, and not in plasma, highlighting the significance of EVs in the early prediction of individual responses to iCDK4/6 and disease progression. Overall, this study provides insights of the longitudinal characterization of plasma‐borne EVs in both a healthy group and HR^+^ MBC patients under iCDK4/6 therapies. Combined vesiclemia and EV sphingolipid profile emphasize the promising potential of EVs as non‐invasive biomarkers for monitoring early treatment response.

## 1 INTRODUCTION

Extracellular vesicles (EVs) are lipid bilayer nanoparticles ranging from 40 to 1000 nm in size, released by all cells into the extracellular space and present in various body fluids, including urine, saliva and blood (Colombo et al., 2014; Costa‐Silva et al., 2015; Fujita et al., 2016; Peinado et al., 2011). EVs play a crucial role in cell‐cell communication by transporting a diverse array of proteins, nucleic acids and lipids to recipient cells (Yáñez‐Mó et al., 2015). Recently, there has been increasing focus on the significance of EVs in pathologies such as cancers (Becker et al., 2016; Katsuda et al., 2014; Schwich & Rebmann, 2018; Villagrasa et al., 2014). The cargo of EVs reflects their parent cells and may contribute to multiple aspects of cancer progression. By encapsulating and delivering bioactive molecules, EVs can influence cellular behaviour, modulate the tumour microenvironment, and promote tumour growth, invasion and metastasis (Becker et al., 2016; Hoshino et al., 2015; Peinado et al., 2012; Tkach & Théry, 2016). Moreover, tumour cells release greater quantities of EVs compared to non‐tumourous cells (Cappello et al., 2017; Lehrer et al., 2019). Consequently, EV concentration and composition are emerging as potential biomarkers for continuously monitoring cancer patient outcomes and tumour responses to treatments (Lane et al., 2018; Melo et al., 2015; Øverbye et al., 2015; Redzic et al., 2014; Szajnik et al., 2013).

Lipids associated with EVs, which comprise the primary components of their outer membranes, offer enhanced sensitivity and specificity, as compared to direct plasma lipid analysis (Ghadami & Dellinger, 2023). Lipids play significant roles in cancer development, with ceramides, a class of bioactive sphingolipids, being critical in regulating cell death (Obeid et al., 1993) and survival (Moro et al., 2018). A recent study confirmed that sphingolipids (ceramides and sphingomyelins) are enriched in EVs produced by breast cancer cells, when compared to normal cells (Dorado et al., 2024). Moreover, cells deficient in ceramide production resist chemo‐ and radio‐therapies (Chmura et al., 1997; Sautin et al., 2000), while elevated ceramide levels have been observed in breast cancer tissues compared to the normal ones, positively correlating with disease severity (Schiffmann et al., 2009). Consequently, EV‐associated lipids have garnered significant interest as potential cancer biomarkers. Direct profiling of EV content, alongside EV concentration from patient body fluids, may provide valuable clinical insights and holds promise as a non‐invasive monitoring tool.

Breast cancer is the most prevalent and malignant cancer among women, representing the most frequently diagnosed cancer worldwide, with an estimated 2.26 million cases and approximately 685,000 female deaths recorded in 2020 (Wilkinson & Gathani, 2022). Breast cancer displays molecular and histological heterogeneity and can be classified into three main subtypes: tumours expressing hormone receptors (hormone receptor‐positive [HR^+^]: oestrogen receptor (ER^+^) or progesterone receptor [PR^+^]), tumours expressing human epidermal growth factor receptor 2 (HER2^+^) and triple‐negative breast cancer (TNBC), which lacks ER, PR and HER2 expression (Lehmann et al., 2011; Liedtke et al., 2008). This classification has led to targeted clinical management strategies focusing on the tumour's biology rather than on the tumour burden solely. In metastatic disease, the primary goals are to improve quality of life and survival rates. Notably, 15% to 30% of breast cancer patients may experience relapse with distant metastasis (Miller et al., 2019; Voogd et al., 2001), characterized by a median survival rate of 1 to 4 years (Grinda et al., 2021). In this study, we focused on HR^+^/HER2^−^ metastatic breast cancer (MBC) patients included in the prospective EPICURE cohort (NCT03958136) (Colombié et al., 2021). We selected patients treated with first‐line cyclin‐dependent kinase 4/6 inhibitors (iCDK4/6), such as palbociclib, abemaciclib or ribociclib, in combination with endocrine therapy, as recommended by International clinical guidelines (Cardoso et al., 2020; Gradishar et al., 2020).

Currently, the prevailing approach for reviewing therapeutic response in MBC patients involves serial imaging (Lima et al., 2019). Liquid biopsies offer an alternate, non‐invasive method for real‐time surveillance of cancer progression and response to treatment, aiding clinical decision‐making. The prevailing circulating biomarker for breast cancer monitoring is the serum level of cancer antigen 15‐3 (CA 15‐3), though its correlation with cancer progression is validated in only about half of the patients (Dawson et al., 2013; Duffy et al., 2010). Additionally, in advanced ER^+^/HER2^−^ breast cancer, a phase 3 trial demonstrated that circulating tumour DNA analysis could serve as a biomarker for detecting b*ESR1* mutations. Switching from aromatase inhibitor and palbociclib treatment to fulvestrant and palbociclib upon detection of rising bESR1 mutations led to significantly improved progression‐free survival (Bidard et al., 2022). Therefore, identifying precise and minimally invasive biomarkers to predict therapeutic response of MBC patients is crucial. In this context, EVs are gaining increasing interest.

In this study, we aimed at defining whether EVs could serve as circulating biological signs that anticipate the clinical responses in HR^+^ MBC patients to iCDK4/6. We investigated the dynamics of plasma EV concentration (vesiclemia) during the treatment period between patients and in comparison to age‐matched healthy women. Variations in the EV concentration were observed in the cohort of MBC, whereas this parameter remained mainly constant over a 10‐month period in healthy subjects. Notably, an increased trend was noted in the group of iCDK4/6‐resistant patients as early as 2 months following the treatment initiation. Additionally, lipidomic analysis unveiled a distinct sphingolipid profile in EVs from MBC patients compared to healthy subjects. Elevated levels of 16 ceramides and sphingomyelins constituted a discriminative signature, distinguishing patients with poor treatment responses from those with favourable outcomes. Thus, our data suggest that combining EV concentration measurement and sphingolipid cargo analysis could be useful as monitoring tools in HR^+^ MBC patients treated with iCDK4/6. Additionally, these approaches may have potential as early indicators of tumour progression.

## 2 METHODS

### 2.1 Ethics approval

Blood samples from healthy female subjects used in this project were obtained from the Etablissement Français du Sang (EFS, Nantes, France, agreement n°2023007306) (EPICURE Healthy: #H). MBC patient plasma samples were obtained via the prospective cohort EPICURE bio‐collection (NCT03958136, agreement 2018‐A00959‐46) from the Institut de Cancérologie de l'Ouest, Nantes, Angers, France (ICO). All women participated voluntarily, and their personal data was kept anonymous. Written and signed informed consent were obtained from patients prior to any trial‐related procedure, including sample collection.

### 2.2 Human plasma samples

Blood samples from 45 healthy females (average age was 54 ± 8 years) were collected in EDTA tubes (5 mL) and centrifuged within 4 h at 1500 × *g* for 10 min. For the longitudinal group of healthy female subjects, blood samples were taken 3 times a year for 2 years at EFS. The mean age of healthy women included in the longitudinal study was 46 ± 10 years (*n* = 8). Collected plasma was aliquoted and stored at −80°C.

MBC patient plasma samples were collected from women aged over 18, diagnosed with locally advanced breast cancer at the ICO. Plasma samples were collected at different times (at inclusion then every 2 to 6 months) over a 4‐year period. For each patient, one EDTA tube (5 mL) was collected and centrifuged at 1500 × *g* for 10 min, following a standardized procedure (Colombié et al., 2021). Plasma was aliquoted and stored at −80°C. Included iCDK4/6 HR^+^ patients (ER^+^ and/or PR^+^, and HER2^−^, *n* = 44) were classified according to the anatomical‐pathology results of the metastatic biopsy at the screening of each sequence; their mean age was 60 ± 14 years. HER2^+^ patients, *n* = 15 (average age: 55 ± 16 years old) and TNBC patients, *n* = 8 (average age: 51 ± 9 years old) were included in the screening vesiclemia study. iCDK4/6 HR^+^ MBC patients were considered as treatment‐sensitive if they had not exhibited any clinical progression within 18 months of study inclusion (*n* = 25). Resistant patients showed clinical progression within 6 months of inclusion (*n* = 7). Intermediate patients sensitivity to therapy was defined as disease progression between 6 and 18 months (*n* = 12) (Table 1).

**TABLE 1:** Clinical information of HR^+^ MBC patients.

iCDK4/6 resistant patients (*n* = 7)					Lipid analysis							
**Biobank**	**Age**	**Elapsed time to progression (months)**	**Immediately metastatic**	**1**st **therapeutic line**	**Visit 1**	**Follow up**	**Vesiclemia measurement**	**Histopathology**	**Histological grade**	**ER**	**PR**	**HER2**
#029	60	6	NO	Abemaciclib^a^ + Fulvestrant	x	x	x	NST	III	100	5	+
#048	55	6	NO	Abemaciclib^a^ + Fulvestrant	x		x	ILC	II	100	20	neg
#056	52	6	NO	Ribociclib^a^ + Fulvestrant	x	x	x	NST	II	100	100	neg
#072	56	2	NO	Ribociclib^a^ + Fulvestrant	x	x	x	NST	III	95	75	neg
#159	53	5	YES	Ribociclib^a^ + Letrozole + LH‐RH agonists	x		x	NST	II	40	neg	neg
#160	52	2	YES	Ribociclib^a^ + Letrozole	x		x	ILC	II	100	100	neg
#206	56	6	NO	Abemaciclib^a^ + Letrozole	x		x	NST	NA	100	70	neg

**iCDK4/6 intermediate patients (** *n* ** = 12)**					**Lipid analysis**							
**Biobank**	**Age**	**Elapsed time to relapse (months)**	**Immediately metastatic**	**1^st^ therapeutic line**	**Visit 1**	**Follow up**	**Vesiclemia measurement**	**Histopathology**	**Histological grade**	**ER**	**PR**	**HER2**
#017	59	9	YES	Abemaciclib^a^ + Letrozole	x	x	x	ILC	II	100	20	neg
#027	73	18	NO	Palbociclib^a^ + Anastrozole	x	x	x	NST	II	90	70	neg
#041	76	14	NO	Palbociclib^a^ + Letrozole	x	x	x	NST	NA	95	95	+
#049	61	17	NO	Abemaciclib^a^ + Fulvestrant			x	NST	III	100	15	neg
#060	74	18	NO	Palbociclib^a^ + Letrozole	x		x	NST	II	100	40	neg
#063	50	11	NO	Abemaciclib^a^ + Fulvestrant	x		x	NST	III	100	100	+
#079	40	11	NO	Abemaciclib^a^ + Letrozole + LH‐RH agonists	x		x	NST	III	100	10	+
#080	68	9	NO	Palbociclib^a^ + Fulvestrant			x	NST	II	100	neg	+
#084	65	10	YES	Abemaciclib^a^ + Letrozole	x		x	NST	II	100	100	neg
#105	74	14	NO	Ribociclib^a^ + Letrozole	x		x	NST	I	100	100	neg
#190	72	9	YES	Ribociclib^a^ + Letrozole	x		x	ILC	II	100	90	neg
#193	28	7	YES	Ribociclib^a^ + Letrozole + LH‐RH agonists	x		x	NST	III	100	60	neg

**iCDK4/6 sensitive patients (** *n* ** = 25)**					**Lipid analysis**							
**Biobank**	**Age**	**Elapsed time to relapse (months)**	**Immediately metastatic**	**1^st^ therapeutic line**	**Visit 1**	**Follow up**	**Vesiclemia measurement**	**Histopathology**	**Histological grade**	**ER**	**PR**	**HER2**
#002	57	57	NO	Palbociclib^a^ + Letrozole			x	NST	II	NA	NA	NA
#004	35	56	YES	Palbociclib^a^ + Letrozole + LH‐RH agonists			x	NST	III	90	90	+
#008	42	58	NO	Palbociclib^a^ + Letrozole + LH‐RH agonists			x	NST	II	80	30	neg
#011	58	56	YES	Palbociclib^a^ + Letrozole			x	NST	I	95	50	neg
#012	43	23	YES	Abemaciclib^a^ + LH‐RH agonists + Letrozole			x	NST	II	100	65	neg
#019	55	52	YES	Palbociclib^a^ + Letrozole + Gosereline			x	NST	II	100	70	neg
#020	68	19	NO	Abemaciclib^a^ + Fulvestrant			x	NST	II	100	10	neg
#021	76	54	NO	Palbociclib^a^ + Letrozole			x	NST	II	60	40	neg
#022	69	37	NO	Palbociclib^a^ + Letrozole			x	NST	II	100	60	++
#025	72	53	YES	Palbociclib^a^ + Letrozole			x	NST	II	90	85	neg
#033	44	22	YES	Abemaciclib^a^ + Letrozole + LH‐RH agonists			x	NST	II	95	95	+
#034	84	50	NO	Palbociclib^a^ + Letrozole			x	ILC	I	100	100	neg
#036	63	48	NO	Palbociclib^a^ + Letrozole			x	NST	I	100	100	+
#038	64	42	NO	Abemaciclib^a^ followed by Palbociclib^a^ + Letrozole			x	NST	II	90	100	neg
#040	74	48	YES	Palbociclib^a^ + Letrozole			x	NST	III	100	neg	+
#043	75	42	NO	Ribociclib^a^ + Fulvestrant	x	x	x	NST	II	100	70	neg
#044	50	34	NO	Ribociclib^a^ + Letrozole	x	x	x	NST	II	100	90	neg
#064	34	34	YES	Ribociclib^a^ + Letrozole + LH‐RH agonists followed by Anastrozole	x			NST	II	80	100	neg
#067	66	43	NO	Ribociclib^a^ + Letrozole	x		x	NST	NA	100	40	+
#087	46	28	NO	Ribociclib^a^ + Letrozole + LH‐RH agonists	x		x	NST	II	100	40	neg
#091	83	21	YES	Palbociclib^a^ + Letrozole	x	x	x	NST	II	100	80	neg
#103	72	35	NO	Ribociclib^a^ + Letrozole	x		x	ILC	II	NA	NA	NA
#125	75	30	NO	Abemaciclib^a^ + LH‐RH agonists	x		x	NST	II	100	neg	neg
#137	65	22	NO	Ribociclib^a^ + Letrozole	x		x	NST	I	NA	NA	NA
#139	65	28	NO	Ribociclib^a^ + Letrozole	x		x	NST	I	100	70	NA

^a^ iCDK4/6.

Abbreviation: ILC, invasive lobular carcinoma; NST, non‐special type carcinoma.

### 2.3 Plasma extracellular vesicles enrichment

Extracellular vesicles (EVs) were separated from plasma by size exclusion chromatography (SEC), using resin columns (qEVoriginal / 70 nm Gen 2) associated with an automatic fraction collector (AFC, Izon Science), according to the manufacturer's protocol. Briefly, plasma samples (500 µL) were thawed on ice for 30 min, then centrifuged at 10,000 × *g* for 20 min (4°C) to remove cellular debris and large particles, before loading on SEC columns to elute particles from different sizes. Fractions 7, 8, 9, 10 and 11, each measuring 400 µL, were collected immediately after the dead volume (dV, 2.9 mL) and eluted in 0.22 µm‐filtered PBS for the optimization of the enrichment protocol. For the main study, only the EV‐enriched fractions corresponding to fractions 7 and 8 were combined into a single 800 µL tube, whereas following fractions (i.e., ranging from fractions 9 to 11) were discarded (Sabbagh et al., 2021).

For Cryo‐TEM experiments, fractions 7–8 were concentrated by ultracentrifugation at 100,00 × *g* for 2 h at 4°C using OPTIMA MAX‐XP ultracentrifuge with MLA‐130 fixed‐angle rotor (Beckman Coulter). Pellets were resuspended in 100 µL of 0.22 µm‐filtered PBS.

For lipidomic analysis, OptiPrep top to bottom density gradient of 5% to 40% was further performed after SEC on 12 mL open‐top polyallomer tubes (Beckman Coulter), as described previously (André‐Grégoire et al., 2018). Fractions of 1 mL were collected from top to bottom and washed in 0.22 µm‐filtered PBS for 3 h at 100,000 × *g*, 4°C, with Beckman Coulter Ultracentrifuge using SW‐41 Ti rotor. Pellets were resuspended in 100 µL of ammonium bicarbonate 50 mM. We have submitted all relevant data of our experiments to the EV‐TRACK knowledgebase (EV‐TRACK ID: EV240035).

### 2.4 Quantification of nanoparticles

The concentration of EVs was measured using single particle tracking (Interferometry Light Microscopy, VideoDrop, Myriade), measuring particle concentration in a real‐time nanometre‐scale optical method (detected range 80–500 nm). Technical replicate by serial dilutions were performed. Alternatively, the concentration was verified through a second and complementary single‐particle tracking procedure, tunable resistive pulse sensing (TRPS), using qNano gold apparatus (Izon Science). The concentration of EVs was determined using np100 nanopore (detected range 50–330 nm) as described previously (André‐Grégoire et al., 2018).

### 2.5 Cryo‐electron microscopy

EVs were separated and concentrated from 500 µL of individual healthy and MBC patient plasma sample as described above, and deposited on glow‐discharged electron microscope grids, followed by vitrification and rapid freezing into liquid ethane using an automatic plunge freezer (EM GP, Leica) under controlled humidity and temperature (*n* = 3 for each). Neither dehydration nor chemical fixatives were used, allowing observation of the EVs in their natural states. EVs were analyzed by cryo‐transmission electron microscopy (cryo‐TEM) at the Microscopy Rennes Imaging Center (MRic, Université de Rennes 1, France) using 200 kV Tecnai G2 T20 Sphera microscope (Thermo Fisher Scientific), equipped with a TemCam XF416 camera (TVIPS) and a single axis cryo‐holder model 626 (Gatan Microscopy, Pleasanton, CA, USA). EV size was estimated using ImageJ software v2.0.0‐rc‐38/1.50b.

### 2.6 Immunoblotting

EVs were separated and concentrated from 500 µL of individual plasma samples as described above and the pellet was resuspended in 50 µL of RIPA and incubated for 30 min on ice. Lysates were clarified at 12,000 × *g* for 15 min. Protein concentration was determined by microBCA protein assay kit (Thermo Scientific). 5 µg of proteins were lysed in boiling Laemmli 6× for 5 min before being resolved by Tris‐acetate SDS‐PAGE, transferred onto nitrocellulose membranes (GE Healthcare) and blotted with the following antibodies, at different dilutions: Syntenin (Abcam Ab133267) diluted at 1/1000, CD9 (System Biosciences EXOAB‐CD9A‐1) diluted at 1/500 and ApoA1 (Santa Cruz Sc‐376818) diluted at 1/1000. Membranes were incubated with HRP‐conjugated secondary antibodies (Southern Biotech), diluted at 1/5000. Membranes were revealed by chemiluminescence using HRP substrate (Millipore). Acquisitions were performed with Fusion software, version FX7 16.15 (Vilber Lourmat).

### 2.7 Lipidomic analyses by mass spectrometry

Plasma EVs were enriched by SEC and Optiprep Density Gradient as described above for the untargeted lipid analysis, and only by SEC for the targeted lipid analysis. Lipids were extracted from 200 µL of EV samples by the methyl‐tert‐butyl ether (MTBE) method for both untargeted and targeted lipid mass spectrometry analyses. EV samples were normalized by vesiclemia for untargeted mass spectrometry, performed by liquid chromatography‐high‐resolution mass spectrometry as extensively described (Croyal et al., 2018; Durand et al., 2021; Kaabia et al., 2018; Matyash et al., 2008). Sphingomyelins, ceramides and triacylglycerols (TG) were further quantified in 200 µL of EV purified by SEC and plasma samples by a targeted lipid liquid chromatography‐tandem mass spectrometry (LC‐MS/MS) as described previously (Alvarez‐Dorta et al., 2018; Croyal et al., 2018; Darabi et al., 2023; Lallement et al., 2023). Plasma apolipoproteins were determined by liquid chromatography‐tandem mass spectrometry, as described previously (Welsh et al., 2024). The methodological details can be found in Extended Data Method.

### 2.8 Statistical analysis

Data are expressed as mean ± S.E.M. and are representative of at least three independent experiments unless otherwise stated. Statistical analysis was performed with GraphPad Prism8.3.0 software using unpaired two‐tailed Mann–Whitney *U*‐test (non‐parametric test), parametric Student test or ordinary one‐way analysis of variance (ANOVA). For each statistical test, a *p*‐value inferior to 0.05 was considered significant. We analyzed concentration variations between T0, T4, and T8 months in eight healthy subjects and calculated the coefficient of variation for each subject. We obtained a mean coefficient of variation of 0.43 with a standard deviation of 0.19. Based on this result, with a 95% confidence interval (the estimated mean ± 1.96 times the standard deviation), we considered a vesiclemia increase over 181% significant. The proportion of each lipid species was calculated by first averaging the concentrations for each patient within their respective groups. Then, the average concentration for each patient group was divided by the overall average concentration of all patients. Thus, each point represents a lipid species. The concentration of lipids in individuals was analyzed using a multivariate principal component analysis (PCA). The log‐rank test was used for the analysis of the Kaplan–Meier curve. To determine the “EV‐sphingo score”, threshold values were determined with treatment‐sensitive patients (*n* = 10), by averaging the concentrations of each of the 16 lipids and adding the standard deviation to these values. A lipid score was considered elevated if more than 3 out of 16 lipid species exceeded these threshold values. Logistic regression classification with recursive feature elimination (LR‐RFE) analysis was performed for the selection of a subset of the most relevant features for the analysis between groups (i.e., resistant and sensitive patients). To evaluate the performance of the LR‐RFE model, leave‐one‐individual‐out cross‐validated LR classification was performed. The Area Under the Receiver Operating Characteristic (AUROC) curve was determined to measure the accuracy of the LR classification models generated with a reduced number of features.

## 3 RESULTS

### 3.1 SEC‐based EV enrichment is suitable for the analysis of plasma particles

**FIGURE 1:**
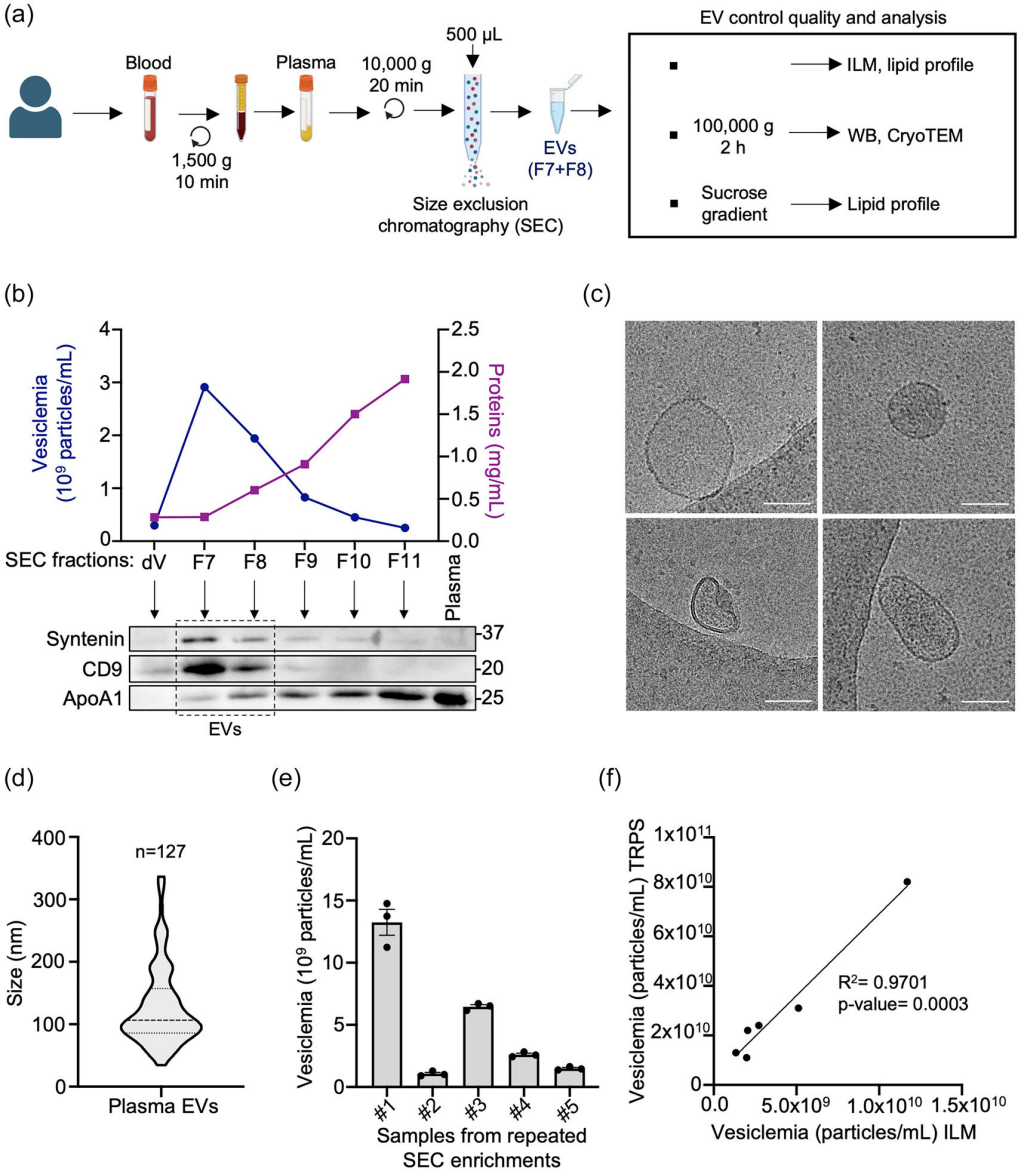
Enrichment and characterization of circulating EVs in plasma. (a) Plasma‐derived EV enrichment protocol. (b) Plasma EV concentration (vesiclemia) measured by VideoDrop (ILM) and protein concentration per SEC fractions in one healthy donor (top). Immunoblotting of SEC fractions from an individual plasma sample (after ultracentrifugation) using EV marker antibodies anti‐syntenin and anti‐CD9, and lipoproteins marker anti‐ApoA1 antibody (bottom). This is representative of *n* = 3 individual samples. (c) Cryo‐TEM images of plasma EVs from a healthy donor (bar scale = 100 nm). This is representative of *n* = 3 individual samples. (d) EV size estimated by Cryo‐TEM, (*n* = 127, mean EVs size: 128 nm ± 64 nm). (e) Repeated EV isolation (*n* = 3) of five different healthy donor plasma samples, and concentration determined with ILM. (f) Comparison of plasma EV concentration measured with ILM versus qNano (TRPS) (*n* = 6). All panels are representative of at least three independent experiments unless otherwise stated, Student test and ANOVA, ^*^ *p* < 0.05, ^**^ *p* < 0.01. Cryo‐TEM, cryo‐transmission electron microscopy; EV, extracellular vesicle; ILM, interferometric light microscopy; SEC, size exclusion chromatography; TRPS, tunable resistive pulse sensing.

In the last decade, efforts to standardize EV enrichment protocols have been undertaken, though they still exhibit variations in cost, required equipment, processing duration and input material volume (Théry et al., 2018). The challenge lies in devising a protocol tailored to encompass EV characteristics. With the aim of future clinical applications, we established an EV enrichment protocol starting from 500 µL of frozen human plasma. Plasma, isolated from whole blood, underwent debris removal before being loaded onto automated size‐exclusion chromatography (SEC, qEV isolation columns, Figure 1a). Additional steps were performed for certain analyses to discard protein aggregates and lipoprotein particles, involving ultracentrifugation (UC) and iodixanol density gradient methods.

Following SEC, vesicle concentration in plasma (vesiclemia) was estimated using interferometric light microscopy (ILM), a rapid and straightforward technique requiring only a few microliters of sample volume (Sabbagh et al., 2021). Vesiclemia, along with protein concentration and content, were analyzed across serial eluted SEC fractions. The highest levels of CD9 and Syntenin EV markers were observed in ultra‐centrifugated fractions 7 and 8, while apolipoprotein A‐1 (ApoA1), indicative of high‐density lipoproteins (HDL), predominated in subsequent fractions (Figure 1b). Upon SEC‐UC separation, the typical morphology of EVs was observed with cryo‐TEM, alongside with minimal protein aggregates (Figure 1c). Diameter measurements via cryo‐TEM ranged from 34 to 336 nm (mean 128 ± 64 nm) (Figure 1d).

To assess the protocol reproducibility, both enrichment and measurement procedures were replicated three times for five randomly tested samples. This demonstrates robust repeatability for each sample, with coefficients of variation ranging from 4.3 to 18.5% (Figure 1e). The reliability of calibration‐free ILM was estimated in comparison to TRPS (qNano). With a correlation coefficient of 0.97 and consistent rankings obtained from six random samples, ILM data aligned with those collected via TRPS (Figure 1f).

Therefore, we devised a protocol that facilitates the analysis of EVs from liquid biopsies, and appears to be efficient, simplified, and clinically adaptable for processing a high number of samples, starting from a minimal plasma volume.

### 3.2 Vesiclemia values vary over time in iCDK4/6 HR^+^ MBC patients compared to healthy donors

**FIGURE 2:**
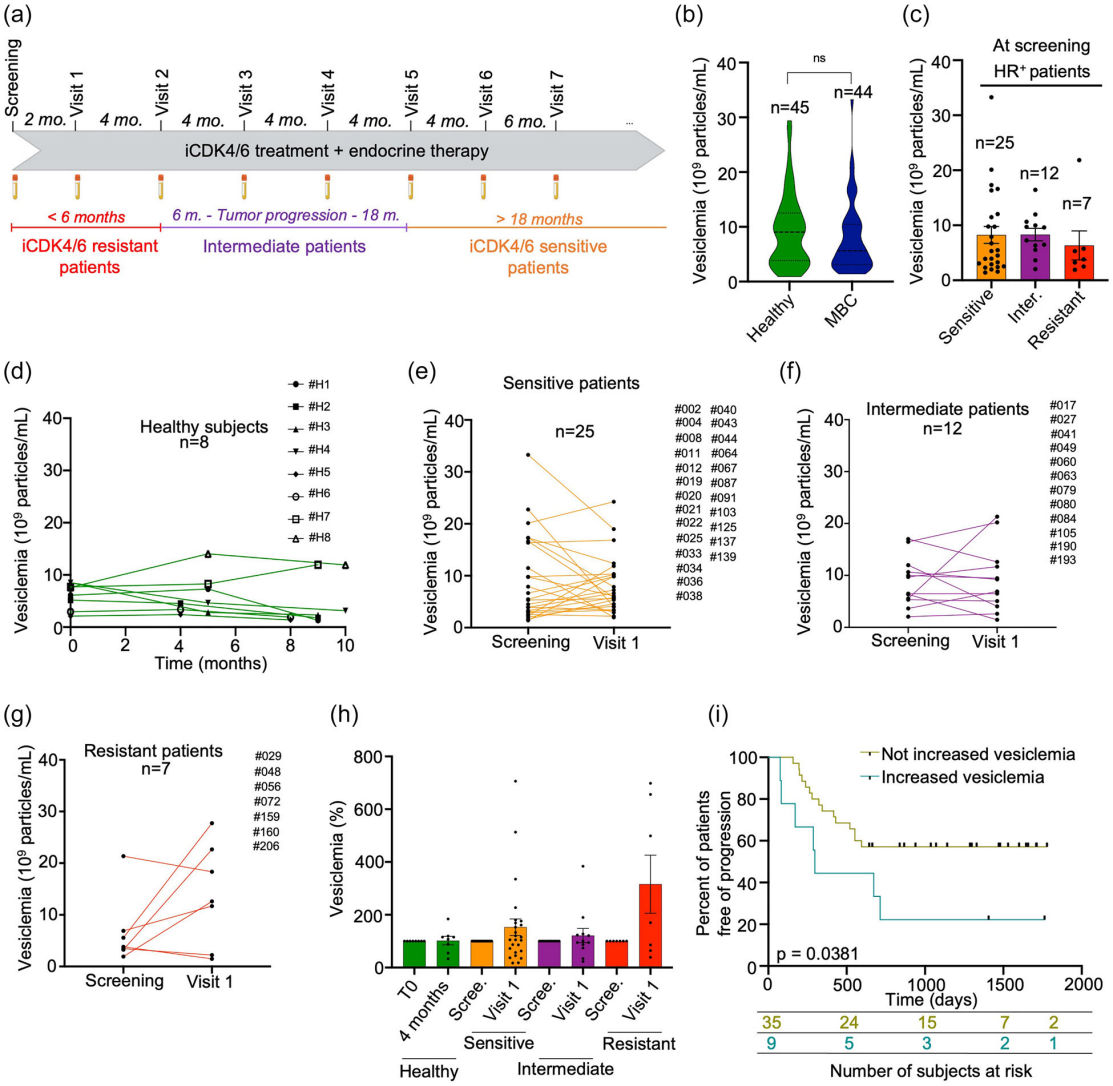
Evolution of plasma EV concentration in HR+ MBC patients. (a) Timeline of sample collection associated with the clinical status of MBC patients from EPICURE cohort. (b) Vesiclemia measured by VideoDrop in healthy donors (*n* = 45, average age: 54 ± 8 years old, average vesiclemia: 9.5 ± 7 × 10^9^ particles/mL) and HR^+^ MBC patients that received iCDK4/6 treatment as first therapeutic line (*n* = 44, average age: 60 ± 14 years old, average vesiclemia: 8.7 ± 7.1 × `10^9^ particles/mL). (c) Vesiclemia measured by VideoDrop in HR^+^ MBC patients according to their clinical status (sensitive *n* = 25, average vesiclemia: 9.3 ± 8.2 × 10^9^ particles/mL intermediate *n* = 12, average vesiclemia: 8.8 ± 4.7 × 10^9^ particles/mL; resistant *n* = 7, average vesiclemia: 6.6 ± 6.7 × 10^9^ particles/mL). (d) Longitudinal vesiclemia monitored in healthy women by Videodrop (*n* = 8, average age: 46 ± 10 years old) over 10 months. (e) Vesiclemia monitored in sensitive patients (*n* = 25) by Videodrop during the 2 first months. (f) Vesiclemia monitored in intermediate patients (*n* = 12) by Videodrop during the 2 first months. (g) Vesiclemia monitoring in resistant patients (*n* = 7) by Videodrop during the 2 first months. (h) Histogram showing vesiclemia (in percent, normalized to screening time) at T0 (screening) and 2 months in healthy subjects (*n* = 8), sensitive (*n* = 25), intermediate (*n* = 12) and resistant patients (*n* = 7). (i) The Kaplan–Meier survival curve for MBC patients illustrates the duration of disease progression (in days) among those who exhibit an increase in vesiclemia (> 181%) from screening to 2 months post‐treatment initiation, as well as those who do not display such increase. The number of patients at risk at 0, 500, 1000, 1500 and 1763 days for each of these 2 groups are shown in the table immediately below the survival curve. For all panels, statistical analyses were performed using the Student test and ANOVA test. ANOVA, analysis of variance; EV, extracellular vesicle; HR^+^, hormone receptor‐positive; MBC, metastatic breast cancer; ns, not significant.

The EPICURE cohort was designed to allow a retrospective analysis of clinic‐biological data from MBC patients, and in coordination with periodic blood sampling (Figure 2a). We initiated our study by collecting the clinical information from 44 HR^+^ MBC patients treated with iCDK4/6 (Table 1). Patients were further categorized in three groups based on their response to the treatment: resistant (i.e., experiencing disease progression within 6 months), intermediate (i.e., exhibiting progression between 6 and 18 months), and sensitive (i.e., showing no tumour progression within at least 18 months) (Figure 2a). Plasma EV concentration was monitored at screening and throughout treatments using frozen plasma obtained during follow‐up visits (Figure S1).

We first launched a comparative analysis of plasma EV concentration at the screening entry point, before MBC dedicated treatment. Deploying the SEC‐ILM procedure, there were no significant differences in the vesiclemia values from HR^+^ MBC patients and age‐matched healthy women (Figure 2b), nor with other BC subtypes (i.e., HER2^+^ and TNBC) (Figure S2A). Furthermore, the vesiclemia levels remained unaffected by the first‐line treatment in HR^+^/HER2^−^ patients (Figure S2A). Similarly, little variation in vesiclemia was observed in the three groups hierarchized according to their subsequent response to iCDK4/6 treatment (i.e., sensitive, intermediate and resistant patients) (Figure 2c). In addition, cryo‐TEM characterization of plasma EVs confirmed a heterogeneous population, with similar morphological features between the patient groups and the healthy subjects (Figure S2B). Finally, we did not find any difference in the vesicle count at the screening phase between patients with de novo and recurrent MBC, suggesting that treatment history may not influence the initial values (Figure S2C).

To further assess the evolution of vesiclemia over time, a prospective analysis was conducted in *n* = 44 HR^+^ MBC patients over a period of 30 months (Figure S1). A similar approach was employed in a cohort of 8 age‐matched healthy women over a period of 10 months, revealing that vesiclemia values remained mostly steady in this heathy cohort (Figure 2d). To next evaluate whether EVs quantity may reflect the patient responses to iCDK4/6, particle counts at screening were compared with those from the first evaluation visit, that is around 2 months post‐treatment initiation (Figure 2e–h). Resistant patients showed a more pronounced increase in vesiclemia at the 2‐month evaluation visit (Figure 2g–h). No noticeable increase was observed at 2 months in patients categorized as sensitive (Figure 2e) or intermediate (Figure 2f). The 44 patients were next separated in two groups: increased vesiclemia by 181% or not‐increased one, at 2 months after screening time for each patient. Interestingly, the Kaplan‐Meier curve revealed an early progression for patients exhibiting an elevated vesiclemia (*p* = 0.0381) (Figure 2i). This suggests that elevated vesiclemia after 2 months may inform on the response to iCDK4/6. However, this single parameter may be insufficient to reliably distinguish between patient groups (sensitive, intermediate or resistant) and predict the disease progression.

### 3.3 Lipidomic analysis of plasma EVs showed significant differences between healthy subjects and HR^+^ MBC patients

**FIGURE 3:**
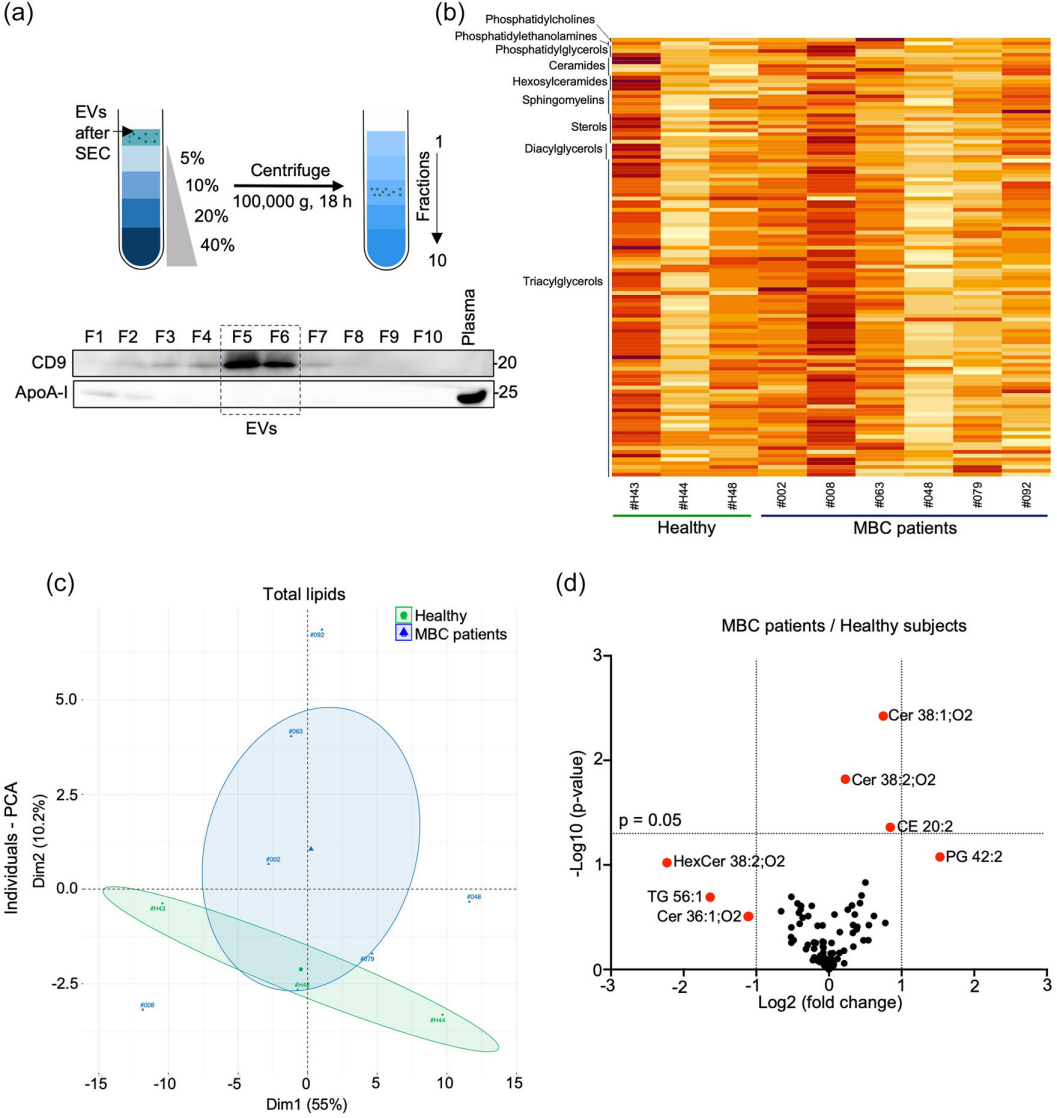
Lipidomic analysis of plasma EVs from healthy subjects and HR+ MBC patients before treatment initiation. (a) Plasma EVs enriched after SEC were loaded on a density gradient. Immunoblotting was performed on Optiprep density gradient fractions, using anti‐CD9 and anti‐apoA‐I antibodies. This is representative of *n* = 3 individual samples. (b) The HeatMap shows the quantification of each one of the 116 lipids per individual (*n* = 3 healthy donors and *n* = 6 MBC patients), by *n* = 9 lipid classes. (c) Principal component analysis represents the untargeted lipid analysis (*n* = 116 lipids) clustered between healthy donors (*n* = 3) and HR^+^ MBC patients (*n* = 6). (d) Volcano plot represents the untargeted lipid analysis (*n* = 116) by mass spectrometry from EV samples enriched after SEC followed by Optiprep density gradient. It shows statistical significance (log10 *p*‐value) and magnitude ((log2 ‐ fold change) of differences in lipid expression profiles between plasma EVs from healthy donors (*n* = 3) and MBC patients at screening time (*n* = 6). Vesiclemia was normalized. CE, cholesteryl ester; Cer, ceramides; EV, extracellular vesicle; HexCer, hexosylceramides; HR^+^, hormone receptor‐positive; MBC, metastatic breast cancer; PG, phosphatidylglycerols; SEC, size exclusion chromatography; SM, sphingomyelins; TG, Triacylglycerols.

To go further, we aimed to characterize the lipid profile associated with plasma EVs from both healthy subjects (*n* = 3) and HR^+^ MBC patients (*n* = 6). To this aim, an untargeted and comparative lipidomic analysis was conducted at the screening time‐point, starting with similar vesicle count, estimated with ILM. To minimize plasma contaminants, such as small‐sized lipoproteins (e.g., HDL, (Welsh et al., 2024)), SEC fractions were further processed with iodixanol density gradients (Figure 3a), resulting in a limited apolipoprotein presence in the CD9‐positive EV‐enriched fractions (Figure 3a and Table 2). The untargeted mass spectrometry‐based lipidomic analysis identified 116 lipid species across 9 lipid classes (Figure 3b, Table S1). Surprisingly, glycerophospholipids were poorly detected in EV samples compared to acylglycerols, likely due to EV sample dilution from SEC enrichment and stringent variable selection thresholds (Extended Data Method). While the presence of acylglycerols may suggest minor lipoprotein contamination, quantification of apolipoproteins indicated minimal pollution (Table 2).

**TABLE 2:** Quantification of apolipoproteins in plasma EVs enriched after SEC and Optiprep density gradient, and in plasma.

	EV									Plasma		
Apolipoproteins (µg/mL)	#H43	#H44	#H48	#002	#008	#063	#048	#092	#079	#H48	#063	#079
ApoA‐I	0.26	0.26	3.26	1.51	–	1.99	1.42	1.37	0.43	1178.03	996.97	1236.52
ApoA‐II	–	–	–	–	–	–	–	–	–	373.03	366.13	346.5
ApoB100	–	–	–	–	–	–	–	–	–	754.12	954.28	771.85
ApoC‐I	–	–	–	–	–	–	–	–	–	12.04	14.62	18.09
ApoC‐II	–	–	–	–	–	–	–	–	–	19.44	10.06	8.83
ApoC‐III	0.03	–	0.07	0.01	–	–	–	–	0.13	91.86	36.68	45.45
ApoE	–	0.38	–	–	–	–	–	–	0.01	22.04	9.59	11.25

*Note*: EV plasma analysis was performed in 3 healthy subjects, 3 sensitive and 3 resistant patients. Plasma analysis was performed in 1 healthy subject, 1 sensitive and 1 resistant patient.

Comparison between healthy subjects and MBC patients for individual lipid species with diverse fatty acyl tail combinations highlighted differences in lipid distribution. PCA illustrated the EV‐associated lipid signature for MBC patients (Figure 3c). Particularly, among the seven differentially expressed lipids, four belonged to the sphingolipid category (Figure 3d).

### 3.4 Elevated EV‐associated sphingolipids as early biomarkers of treatment response following iCDK4/6 initiation in HR^+^ MBC patients

**FIGURE 4:**
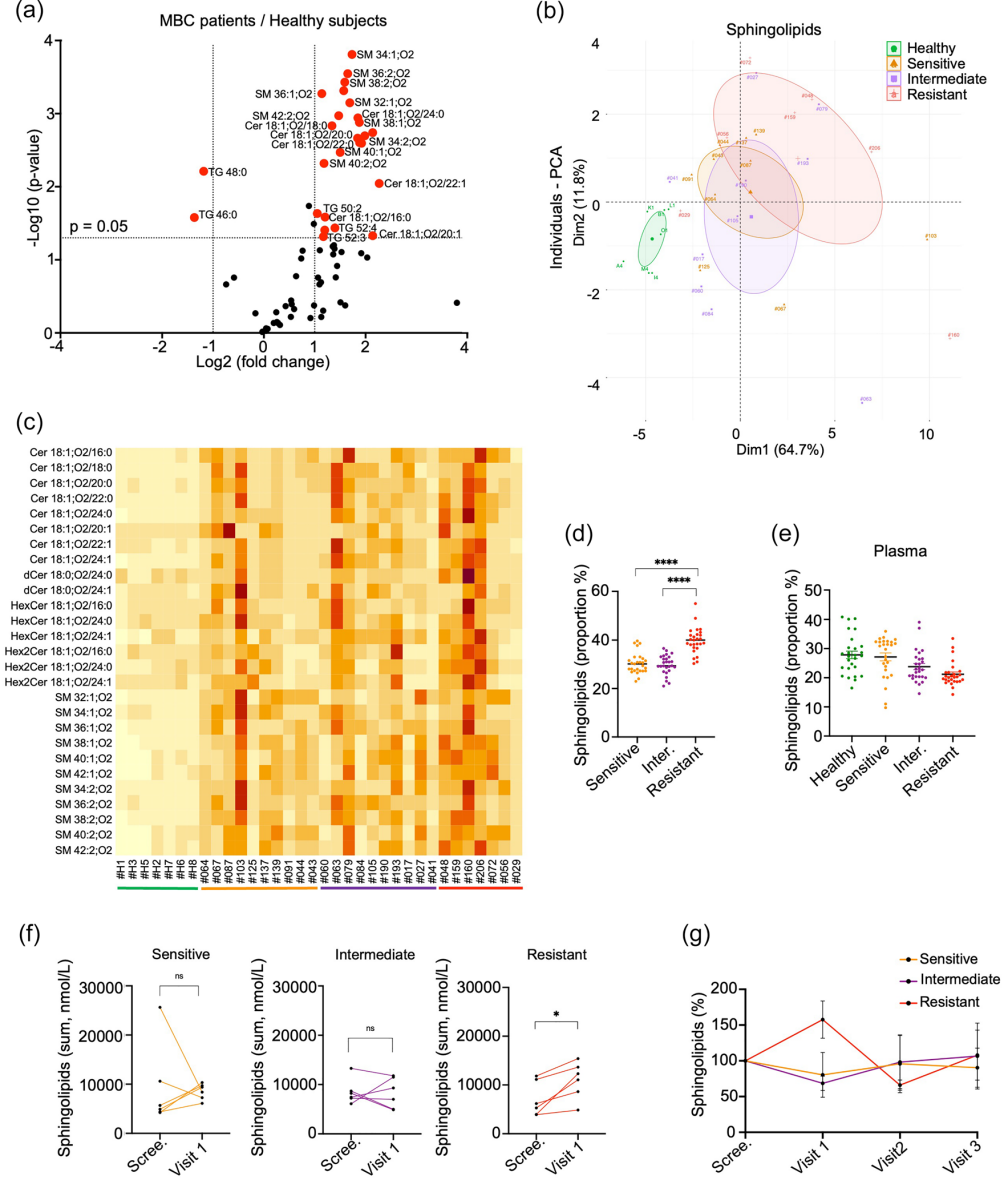
EV‐associated sphingolipids are more abundant in resistant HR^+^ MBC patients 2 months after CDK4/6 inhibitor treatment initiation (visit 1). (a) Targeted analysis of lipids (*n* = 71) by mass spectrometry from EV samples enriched after SEC. The volcano plot shows statistical significance (log10 *p*‐value) and magnitude ((log2 ‐ fold change) of differences in lipid expression profiles between plasma EVs from healthy donors (*n* = 4) and MBC patients at evaluation visit 1 (*n* = 11). (b) Plasma EV samples enriched by SEC were analyzed by quantitative lipidomic from four different groups (healthy subjects *n* = 7, sensitive patients *n* = 10, intermediate patients *n* = 10 and resistant patients *n* = 7). Principal component analysis showing differences in sphingolipid clusters signature (27 species) between each indicated group, and (c) HeatMap representing the intensity of each sphingolipid (*n* = 27) concentration per individual (*n* = 34). (d) Proportion of each one of the 27 sphingolipids detected in EV samples per groups (*n* = 10 sensitive patients, *n* = 10 intermediate patients and *n* = 7 resistant patients). The proportion of each lipid species was calculated by first averaging the concentrations for each patient within their respective groups. Then, the average concentration for each patient group was divided by the overall average concentration of all patients. (e) Proportion of each one of the 27 detected sphingolipids directly in plasma by group (*n* = 3 healthy subjects, *n* = 3 sensitive patients, *n* = 5 intermediate patients and *n* = 3 resistant patients). The proportion of each lipid species was calculated by first averaging the concentrations for each patient within their respective groups. Then, the average concentration for each patient group was divided by the overall average concentration of all patients. (f) Concentration of EV‐associated sphingolipids (nmol/L) at screening time and visit 1 in resistant patients (*n* = 6), intermediate patients (*n* = 6) and resistant patients (*n* = 6). (G) Longitudinal analysis of EV‐associated sphingolipids (in percent, normalized to screening time), at screening, visit 1, visit 2 and visit 3 of sensitive patients (*n* = 3), intermediate patients (*n* = 3) and resistant patients (*n* = 3). All panels are representative of at least three independent experiments unless otherwise stated, Student test and ANOVA, ^*^ *p* < 0.05, ^**^ *p* < 0.01, ^***^ *p* < 0.001, ^****^ *p* < 0.0001. ANOVA, analysis of variance; EV, extracellular vesicle; HR^+^, hormone receptor‐positive; MBC, metastatic breast cancer.

To further investigate EV‐associated lipids that might separate iCDK4/6‐resistant patients from better responders, lipidomic analysis was performed in EVs obtained from plasma at visit 1 (approximately 2 months after screening and treatment initiation). This time, a targeted and quantitative mass spectrometry‐based approach was employed on SEC only‐separated EV fractions, and their paired whole plasma. Again, minimal apolipoprotein contamination was seen in the SEC fractions (Table 3). Consistently, we primarily focused on sphingolipids and TG. This analysis quantified 71 EV‐associated lipids, comprising 27 sphingolipids and 44 TG (Figure 4a). PCA and HeatMap showed that EV samples from resistant patients formed a distinct cluster (Figure 4b,c), highlighting the specificity of sphingolipid profiles within each group. In contrast, the TG signature did not exhibit such differences among the patient groups (Figure S3A,B).

**TABLE 3:** Quantification of apolipoproteins in plasma EVs enriched after SEC.

	Sensitive			Intermediate			Resistant		
Apolipoproteins (µg/mL)	#091	#044	#043	#017	#027	#041	#029	#056	#072
ApoA‐I	4.43	4.40	4.17	4.01	4.52	3.94	4.64	4.42	4.89
ApoA‐II	–	–	–	0.05	0.06	–	0.03	0.09	0.14
ApoB100	15.68	–	15.67	–	–	–	–	15.74	–
ApoC‐I	0.03	–	–	0.02	0.09	–	0.02	0.14	–
apoC‐II	–	–	–	–	–	–	–	–	–
apoC‐III	–	–	–	–	–	–	–	–	–
apoE	0.39	0.18	0.32	0.41	0.66	0.20	0.12	0.91	0.14

*Note*: Analysis was performed in 3 sensitive, 3 intermediate and 3 resistant patients at visit 1.

Focusing on EV‐associated sphingolipids at 2 months post‐iCDK4/6 treatment, a higher proportion of the 27 identified sphingolipids was denoted in resistant patients, as opposed to intermediate or sensitive patient categories (Figure 4d). This signature was not seen in the analysis neither at the screening point (Figure S3C), nor within plasma (Figure 4e), thereby highlighting the importance of analyzing sphingolipids associated with EVs rather than from bulk plasma.

Next, we compared these lipids at visit 1 with those at the screening time point. There was a significant increase at visit 1 in the resistant patient group (Figure 4f). This was even further evident with a longitudinal analysis, where the portion of sphingolipids peaked at the 2‐month time point only in resistant patients, before reaching initial baseline (Figure 4g). This latter normalization in the values might be linked to therapeutic regimen switch once clinical progression has been established (Figure S1). These results suggest that changes in EV‐associated sphingolipids can be detected after the initiation of iCDK4/6 treatment, indicating that lipidomic profiling of plasma EVs may help predicting early treatment response in HR^+^ MBC patients.

### 3.5 Ceramide‐sphingomyelin signature in plasma EVs is indicative of the response to CDK4/6 inhibitors in HR^+^ MBC patients

**FIGURE 5:**
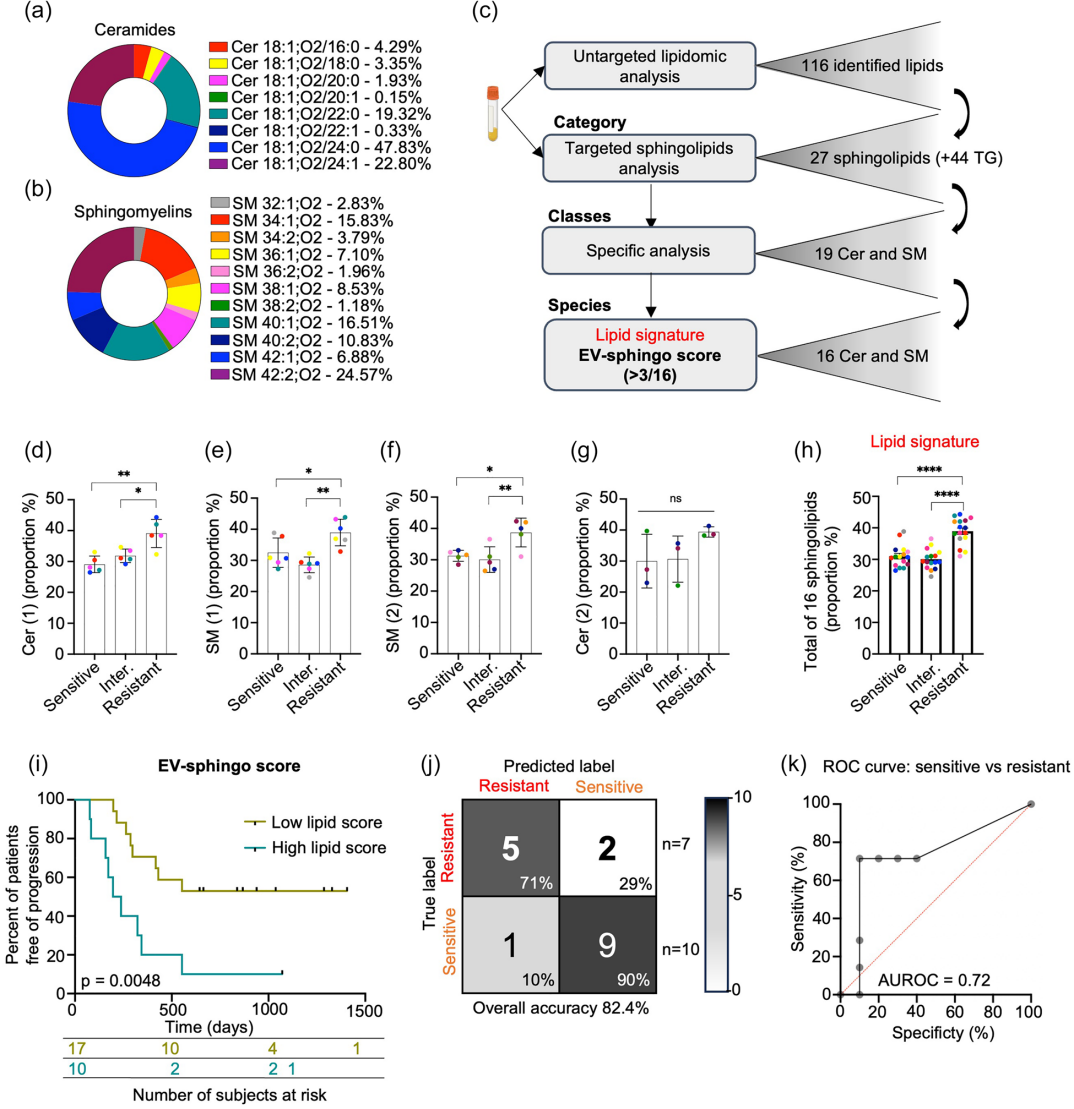
The level of ceramides and sphingomyelins associated with EVs at the first visit is an indicator of response to treatment. (a) Proportion of each ceramide (*n* = 8) quantitatively analyzed on *n* = 27 EV samples from MBC patients. (b) Proportion of each sphingomyelin (*n* = 11) quantitatively analyzed on *n* = 27 EV samples from MBC patients. (c) Representative diagram of the logical pathway leading to the selection of a signature of 16 sphingolipids. (d) Proportion of each one of the five ceramides (1) per groups (*n* = 10 sensitive patients, *n* = 10 intermediate patients and *n* = 7 resistant patients) in EV samples. Cer (1) denotes ceramides containing one double bond. The proportion of each lipid was calculated by first averaging the concentrations for each patient within their respective groups. Then, the average concentration for each patient group was divided by the overall average concentration of all Cer (1). Thus, each point represents a lipid species. (e) Proportion of each one of the six sphingomyelins (1) per groups (*n* = 10 sensitive patients, *n* = 10 intermediate patients and *n* = 7 resistant patients) in EV samples. (f) Proportion of each one of the five sphingomyelins (2) per groups (*n* = 10 sensitive patients, *n* = 10 intermediate patients and *n* = 7 resistant patients) in EV samples. (g) Proportion of each one of the three ceramides (2) per groups (*n* = 10 sensitive patients, *n* = 10 intermediate patients and *n* = 7 resistant patients) in EV samples. (h) Proportion of each one of the and the 5 Cer (1), 6 SM (1) and 5 SM (2) per groups (*n* = 10 sensitive patients, *n* = 10 intermediate patients and *n* = 7 resistant patients) in EV samples. The proportion of each sphingomyelin was calculated as the ratio of its concentration to the total concentration of the 16 sphingolipids. (i) The Kaplan–Meier survival curve for MBC patients illustrates the duration of disease progression (in days) among those who exhibit a high lipid score (> 3/16) 2 months post‐treatment initiation, as well as those who exhibit a low lipid score (≤3/16). (j) Confusion matrix of EV‐associated sphingolipid classification with cross‐validation of sensitive and resistant patient samples showing an overall accuracy of 82.4%. The colour scheme of the confusion matrix is driven by the number of observations rather than percentages. (k) AUROC for the combination of the 16 relevant sphingolipid species was equal to 0.72. All panels are representative of at least three independent experiments unless otherwise stated, Student test and ANOVA, ^*^ *p* < 0.05, ^**^ *p* < 0.01, ^***^ *p* < 0.001, ^****^ *p* < 0.0001. ANOVA, analysis of variance; EV, extracellular vesicle; MBC, metastatic breast cancer.

We next evaluated the relative proportions of the identified sphingolipids (8 ceramides and 11 sphingomyelins, Figure 5a,b). Plasma concentrations fall into the range of expected concentrations (Figure S4A,B, (Quehenberger et al., 2010)). A positive correlation in the total concentrations of the two lipid classes was evidenced in the EVs from 27 patients (*r* = 0.72), (Figure S4C). This correlation was similarly true for plasma‐borne ceramides and sphingomyelins (11 patients, Figure S4D; *r* = 0.71). Given that these two lipid classes share the same biosynthetic pathway, this correlation suggests that the observed differences in sphingolipid synthesis are more likely attributed to class‐specific effects rather than interconversion.

The workflow (Figure 5c) aiming at identifying an EV‐lipid signature linked to the response to iCDK4/6 treatment was initiated with an untargeted analysis unveiling 116 lipid species, among which 4 out of 7 differentially expressed between patients and healthy subjects were sphingolipids. A second targeted analysis resulted in the identification of 27 sphingolipids, including the majority of ceramides and sphingomyelins. From these, three out of four classes were selected based on their overexpression in resistant patients. This was particularly evident for ceramides with one double bond (Cer (1)) (Figure 5d), sphingomyelins with one double bond (SM (1)) (Figure 5e) and sphingomyelins with two double bonds (SM (2)) (Figure 5f). In contrast, the class of ceramides with two double bonds (Cer (2)) did not show significant differences between groups (Figure 5g). These differences were not observed in plasma (Figure S4E–H). The collective overrepresentation of these three classes of sphingolipids, encompassing 16 species in total, was significantly pronounced in resistant patients, as compared to other patient groups (Figure 5h). Notably, Cer 42:1;O2, that is, 18:1;O2/24:0 (Figure S4I) and SM 40:1;O2 (Figure S4J) were more prevalent in the EVs from iCDK4/6 resistant patients compared to the sensitive ones. Again, this was not the case when conducting similar analysis on whole plasma (Figure S4K,L).

Ultimately, we established a predictive treatment response score (“EV‐sphingo score”) based on the sphingolipid signature derived from the 16 species. To calculate this score, threshold values were determined with treatment‐sensitive patients, by averaging the concentrations of each of the 16 lipids and adding the standard deviation to these values (Table S2). We assessed the value of overall accuracy to define the test validity of the score. A lipid score was considered elevated if more than 3 out of 16 lipid species exceeded these threshold values. Patients with a high lipid score demonstrated significantly accelerated disease progression compared to those with a low lipid score (*p* = 0.0048) (Figure 5i). Furthermore, leave‐one‐individual‐out cross‐validation analysis demonstrated that EV samples from resistant patients (*n* = 7) and sensitive patients (*n* = 10) could be classified with an overall accuracy of 82.4% (Figure 5j). Similarly, ROC analysis revealed an area under the ROC curve (AUROC) of 0.72 for the “EV‐sphingo score” (Figure 5k), suggesting its promising potential for use in association with other diagnostic methods, such as PET scans, for a more comprehensive assessment of iCDK4/6 treatment response and to better inform treatment options.

Thus, the EV‐associated sphingolipid profile, specifically ceramides and sphingomyelins, may help predict the failure of iCDK4/6 treatment in HR^+^ MBC patients as early as 2 months after treatment initiation, in comparison to the current 6 months clinical validation. Upon further validation, this may impact clinical treatment strategies.

## 4 DISCUSSION

Liquid biopsy, a relatively non‐invasive method, provides a straightforward strategy for monitoring cancer patients undergoing treatment. EVs emerged as potential indicators of physiological and pathological states (Yuana et al., 2013). However, due to their heterogeneity in terms of size, content and origin (Kalluri & LeBleu, 2020), obtaining EV samples with high yield and purity remains technically challenging. Various isolation techniques were developed, including differential ultracentrifugation, ultrafiltration, SEC, density gradient ultracentrifugation and immunoaffinity isolation. However, isolating EV from plasma remains difficult due to the presence of confounding lipoproteins and highly concentrated proteins (Muller et al., 2014; Simonsen, 2017). Our study revealed that the elevation in the vesicle count is associated with disease progression in MBC patients, and highlighted an EV‐associated sphingolipid profile in resistant patients. As early as 2 months post‐treatment, a signature composed of 16‐ceramide and sphingomyelin may effectively identify patients who are resistant to iCDK4/6 therapy.

In order to enable rapid and convenient processing suitable for clinical applications, EV fractions were enriched through automated SEC, starting from as low as 500 µL of plasma. In this study, we were able to generate vesiclemia data and lipidomic analyses from a single 500 µL sample, which is an advantage for scaling up these data to clinical cohorts. However, a limitation of this approach is to use only the SEC technique, which does not exclude the likely lipoprotein contamination of our EVs samples. For further lipidomic analyses, SEC was combined with density gradient ultracentrifugation, as this dual approach effectively separated chylomicrons and very‐low‐density lipoproteins from EVs based on their size and density characteristics, while also reducing the presence of protein aggregates (Brennan et al., 2020). As a result, this procedure proves to be a reliable method for isolating EVs within complex biological samples. This approach is particularly advantageous for studying heterogeneous EV populations, especially in a pathological context.

Furthermore, conducting studies on EV concentration presents challenges related to both isolation and quantification methods (EV‐TRACK Consortium et al., 2017). Common techniques for quantification include nanoparticles tracking analysis (NTA), TRPS and flow cytometry (FC). In our study, we opted for ILM due to its rapidity, robust reproducibility and suitability for handling a large number of samples. Johnsen et al., summarized that EV concentrations from blood samples of healthy individuals vary across a range of over seven orders of magnitude, with a geometric mean of approximately 1 × 10^10^ particles/mL using different isolation methods (Johnsen et al., 2019). In our study, we measured an approximate baseline vesiclemia of 0.95 × 10^10^ particles/mL in healthy individuals using SEC isolation combined with ILM quantification (Figure 2b). Importantly, by using a control group that sex‐ and age‐matched the MBC group, we provided a longitudinal assessment of plasma EV concentration over a 10‐month period (with three blood draws per donor), demonstrating the intra‐individual stability of these measurements.

Circulating biomarkers are of growing interest due to their potential to assist in the diagnosis and monitoring of cancers at early stages. An overall elevation in vesiclemia was previously documented in various types of cancers (Cappello et al., 2017; Muller et al., 2014; Verma et al., 2015). In breast cancer patients, an increase in the number of EVs has been reported in plasma from stage I to IV compared to healthy individuals (Galindo‐Hernandez et al., 2013). A similar rise was established in the blood of women with TNBC (Stevic et al., 2018). These findings indicate a potential application of EV monitoring in aggressive breast cancers. However, the comparison of circulating EV quantities at various stages of the disease and their correlation with treatment response requires further investigation. In this study, we conducted a longitudinal assessment of EV concentration in the blood plasma of MBC patients. Surprisingly, no overt changes were detected among HR^+^ patients, at the initial screening phase, when compared to healthy subjects. This may be due to the heterogeneity of patient profiles at the metastatic stage, with previous breast cancer history and disparate therapeutic treatments (Figure S1). Consequently, we focused on HR^+^ patients receiving frontline iCDK4/6 combined with endocrine therapy (EPICURE cohort). Notably, a stable plasma EV concentration was observed in patients with no progression within 18 months after treatment initiation. By contrast, a trend towards increased vesiclemia was detected among resistant patients, that is, those with disease progression within the first 6 months post‐treatment. Furthermore, an increase in vesiclemia 2 months post‐iCDK4/6 treatment initiation was associated with accelerated disease progression. Hence, this study represents a significant effort in dissecting the longitudinal evolution of plasma EV concentration in the context of MBC. It highlights the potential of vesiclemia as a promising guide for disease monitoring. Future work will be required to assess and confirm our findings, particularly in other breast cancer subtypes.

EVs contain a wide variety of proteins, sugars, lipids and nucleotide‐based materials such as DNA, mRNA and non‐coding RNAs (Henderson & Azorsa, 2012; Théry et al., 2002). There are three publicly accessible databases cataloguing proteins, nucleic acids and lipids in EVs: ExoCarta, Vesiclepedia and EVpedia (Kalra et al., 2012; Kim et al., 2013; Mathivanan & Simpson, 2009; Mathivanan et al., 2012; Simpson et al., 2012). The lipid composition of EVs reflects their cellular origin and the processes involved in their formation (Ghadami & Dellinger, 2023). The main lipids detected in EVs include cholesterol, sphingomyelins, phosphatidylcholines and phosphatidylserines, and the composition varies depending on the cell of origin (Llorente et al., 2013). In this context, our study identified distinct lipid compositions in plasma‐derived EVs from healthy individuals and MBC patients. Additionally, EV‐based lipid profiles differ in patients with favourable responses to iCDK4/6 from those with confirmed clinical progression. Specifically, we identified two classes of sphingolipids—ceramides and sphingomyelins—that could serve as early indicators of resistance to iCDK4/6 in HR^+^ MBC patients.

Here, we discovered that a panel of 16 lipids (comprising ceramides and sphingomyelins) enabled accurate classification of resistant patients within just 2 months of initiating iCDK4/6 treatment. Among the 7 resistant patients analyzed, 5 were correctly classified, with only one misclassification observed among the 10 sensitive patients assessed. Furthermore, our quantitative lipidomic approach identified enriched EVs‐associated sphingolipids, while their correlation with patient responses to iCDK4/6 underscores the potential of theses lipids as toolbox for monitoring breast cancer and patient stratification. Furthermore, we identified Cer 18:1;O2/24:0 and SM 40:1;O2 in EVs isolated from iCDK4/6‐resistant MBC patients. Of note, this ceramide was previously reported to be upregulated in colorectal cancer (Ecker et al., 2021; El Hindi et al., 2024). The absence of this lipid signature in direct plasma further emphasizes the importance of studying EV‐associated lipids. Previous research by Kar *et al.*, identified a sphingolipid signature distinguishing luminal and TNBC subtypes, as well as stage II and III tumours (Kar et al., 2023). Additionally, Nishida‐Aoki et al., described a differential lipid compositions between EVs from high and low‐metastatic cell lines (Nishida‐Aoki et al., 2020). Furthermore, levels of SM 16:1;O2 have been found to be inversely correlate with mammographic density in breast cancer (His et al., 2021). A recent study has shown that EV circulating in breast cancer patients’ blood are promising source of lipid biomarkers for breast cancer detection and that specific lipid class and species could distinguish primary and MBC (Dorado et al., 2024).

Exploring the functional role of these lipids associated with EVs could provide insights into intercellular communication during oncogenesis. Despite their low abundance, ceramides and sphingomyelins, prominent members of the sphingolipid category, are known to influence EV biogenesis and release, as well as various cancer‐related processes such as apoptosis, cell proliferation and migration (Chiricozzi et al., 2018; Gault et al., 2010; Giussani et al., 2014; Giussani et al., 2014; Hannun & Obeid, 2008). Ceramides can be produced through the hydrolysis of sphingomyelins by sphingomyelinases (Gault et al., 2010), while sphingomyelins are synthesized from ceramides by the sphingomyelin synthases (Tafesse et al., 2007). The lipid signature associated with EVs may has been identified in the specific context of iCDK4/6 and might therefore not be applicable for MBC patients undergoing alternative therapies, such as immune checkpoint inhibitors. Future studies will investigate whether this EV‐associated lipid signature co‐occur in MBC patients receiving alternate therapeutic modalities.

## 5 CONCLUSIONS

Overall, circulating EVs hold promise as novel plasma biomarkers for monitoring MBC and anticipating individual responses to CDK4/6 inhibitors. Our study demonstrated that both the plasma concentration of EVs and their EV‐associated sphingolipid profiles can stratify patients according to their responses to treatment. These findings may contribute to the development of a non‐invasive scoring system to assist in evaluating the efficacy of targeted therapies.

## AUTHOR CONTRIBUTIONS

**Mathilde Richard**: Conceptualization (equal); data curation (lead); methodology (lead); writing—original draft (lead). **Rosalie Moreau**: Data curation (supporting); methodology (supporting). **Mikaël Croyal**: Data curation (equal). **Laurent Mathiot**: Resources (supporting); supervision (supporting). **Jean‐Sébastien Frenel**: Resources (supporting); validation (supporting). **Mario Campone**: Supervision (supporting). **Aurélien Dupont**: Data curation (equal). **Julie GAVARD**: Funding acquisition (lead); investigation (equal); project administration (equal); supervision (equal); validation (equal); visualization (equal). **Gwennan Andre‐Gregoire**: Conceptualization (equal); methodology (equal); supervision (equal); validation (equal); visualization (equal). **Laëtitia Guevel**: Conceptualization (equal); data curation (equal); investigation (lead); methodology (lead); project administration (lead); writing—original draft (equal).

## CONFLICT OF INTEREST STATEMENT

The authors declare no conflicts of interest.

## Supporting information



Supporting Information

Supporting Information

Supporting Information

Supporting Information

Supporting Information

Supporting Information
