# Supplementary material for: Monitoring concentration and lipid signature of plasma extracellular vesicles from HR+ metastatic breast cancer patients under CDK4/6 inhibitors treatment
Source: J Extracell Biol. 2024 Dec 17;3(12):e70013. doi: 10.1002/jex2.70013 (PMC11650302; doi:10.1002/jex2.70013)
Supplement: Supplementary file 1 — Supporting Information [file JEX2-3-e70013-s006.docx]

**Untargeted lipidomic analysis –** The lipidomic analysis was performed by liquid chromatography–high resolution mass spectrometry (LC–HRMS). All solvents were LC-MS grade and were purchased from Biosolve (Valkenswaard, The Netherlands). A mixture of seven endogenous standards (Supplemental Table S1) was added to 200 μL of each EV sample and to 200 μL of 6 replicates of a quality control pool (QC) that was constituted by mixing an equal amount of each EV sample. Lipids were then extracted from all samples using 225 µL of ice-cold methanol. Following 10 seconds vortex, 750 µL of ice-cold methyl-ter-butyl ether were added and the mixture was vortexed for 10 seconds. Finally, 188 µL of water were added and vortexed again for 10 seconds. The final mixture was centrifuged (10,000 g; 10 minutes, 4 °C) and 600 µL of supernatant were transferred to LC-MS vial to be evaporated to dryness under a gentle stream of nitrogen (room temperature). Dried samples were reconstituted with 110 µL mixture of acetonitrile/isopropanol/water (65/30/5, v/v/v). Samples were then arbitrarily randomized before analysis. Lipid separation was achieved on an Acquity® H-Class UPLC system (Waters Corporation, Milford, USA) after injection of 10 µL of sample onto an Acquity® CSH C18 column (2.1 mm × 100 mm, 1.7 μm; Waters Corporation) held at 55 °C. The mobile phase was composed of an acetonitrile/water (60/40, v/v) mixture as solvent A and an isopropanol/acetonitrile (90/10, v/v) mixture as solvent B, each containing 10 mM ammonium acetate and 0.1% formic acid. The elution was carried out using a multistep gradient of solvent B in solvent A over 22 min at a constant flow rate of 400 µL/min. The elution started at 40% solvent B, followed by a linear gradient up to 43% solvent B over 2 min. Solvent B was immediately increased up to 50%, and ramped up to 54% over 10 min. Next, solvent B was increased to 70%, then ramped to 99% over 6 min, and maintained at 99% for 2 min. The eluent composition returned to the initial conditions, and the LC column was equilibrated for 2 min. Detection of lipids was then performed by a Synapt G2 HDMS Q-TOF mass spectrometer equipped with a Z-Spray interface for electrospray ionization (ESI) (Waters Corporation). The resolution mode was used to scan ions with mass-to-charge ratios (*m/z*) from 50 to 1,600 at a mass resolution of 25,000 full width half maximum (FWHM) for both positive (ESI+) and negative (ESI−) ionization modes. The ionization settings were as follows: capillary voltage, +3 and -2 kV (ESI+ and ESI-, respectively); cone voltage, 30 V; desolvation gas (N_2_) flow rate, 900 L/h; desolvation gas/source temperatures, 550/120 °C. Leucine enkephalin solution at 2 µg/mL (50% acetonitrile) was infused at a constant flow rate of 10 µL/min in the lockspray channel, allowing for correction of the measured *m/z* throughout the batch (theoretical *m/z* 556.2771 and 554.2616 for ESI+ and ESI−, respectively). Data were collected in the centroid mode at a rate of four spectra per second. Data acquisition and processing of mass spectrometry data, including peak detection, integration, alignment, and normalization (normalization according to the total intensity of all features in the sample), were achieved using MassLynx® and MakerLynx® software (version 4.1, Waters Corporation). Lipid markers were selected from the detected features using an in-house lipidomic database of 474 lipid species built by the use of lipid standards, the exact mass measured, the elemental compositions with a mass error below 5 ppm, the retention times, and the fragmentation patterns as detailed elsewhere [PMID: 28752209]. To evaluate the performance of the analytical system and control changes in sensitivity of the mass spectrometer, the relative standard deviation (RSD, %) was calculated for all peak areas in QC samples. Finally, selected lipid markers having a RSD value higher than 30% and/or that were not detected in all EV samples were not retained for analysis.

**Supplemental Table S1.** Exogenous internal standards.

| **Exogenous internal Standards** | **Final concentration (µmol/L)** | **Ionization mode** |
| --- | --- | --- |
| Cer 18:1;O2/17:0 | 1 | ESI+ / ESI- |
| SM 35:1;O2 | 10 | ESI+ / ESI- |
| LPC 17:1 | 10 | ESI+ / ESI- |
| ^2^H_62_-PC 16:0/16:0 | 10 | ESI+ / ESI- |
| ^2^H_62_-PE 16:0/16:0 | 10 | ESI+ / ESI- |
| ^2^H_5_-TG 16:0/16:0/18:0 | 50 | ESI+ |
| ^2^H_8_-arachidonate | 10 | ESI- |

*Cer, ceramide; SM, sphingomyelin; LPC, lysophosphatidylcholine; PC, phosphatidylcholine; PE, phosphatidylethanolamine; TG, triacylglycerol; ESI, electrospray ionization.*

**Targeted lipidomic analysis: quantification of sphingolipids –** Sphingolipids were analyzed in EV samples by liquid chromatography-tandem mass spectrometry (LC-MS/MS). All solvents were LC-MS grade and purchased from Biosolve. Standard compounds were obtained from Sigma Aldrich (Saint-Quentin Fallavier, France). A pool of standard solutions, including 17 species of ceramides (Cer) and 11 species of sphingomyelins (SM), was prepared and then serially diluted in methanol (Sigma Aldrich, Saint-Quentin Fallavier, France), to obtain seven standard solutions ranging from 5 to 5,000 nM for Cer and 0.1 to 100 µM for SM. Standard solutions and EV samples (200 µL), were extracted as described in the previous section after the addition of Cer (18:1;O2/17:0) and SM (35:1;O2) as internal standard (IS). LC-MS/MS analyses were performed on a Xevo® TQD mass spectrometer with an ESI interface and an Acquity H-Class® UPLC^TM^ device (Waters Corporation). Data acquisition and analyses were performed with MassLynx® and TargetLynx® software, respectively (version 4.1, Waters Corporation). Samples were injected (10 µL) onto an Acquity® BEH C18 column (2.1 × 50 mm, 1.7 µm, Waters Corporation) held at 60 °C. Compounds were separated with a linear gradient of mobile phase B (50% acetonitrile, 50% isopropanol, 0.1% formic, 10 mM ammonium formate) in mobile phase A (5% acetonitrile, 10 mmol/L ammonium acetate, 0.1% formic acid) at a flow rate of 400 μL/min. Mobile phase B was kept constant for 1 min at 40%, linearly increased from 40% to 99% for 4 min, kept constant for 1.5 min, returned to the initial condition over 0.5 min, and kept constant for 2 min before the next injection. Cer, SM and IS were detected by the mass spectrometer with the ESI interface operating in the positive ion mode (capillary voltage, 3 kV; desolvatation gas (N_2_) flow, 650 L/h; desolvatation gas temperature, 450 °C; source temperature, 120 °C). The multiple reaction mode (MRM) was applied as described in Supplemental Table S2. Chromatographic peak area ratios between lipids and their respective IS constituted the detector responses. Standard samples were used to plot calibration curves and a linear regression model (1/x weighting) was used for quantification.

**Supplemental Table S2.** MRM parameters used for sphingolipid detection.

| **Species** | **Cone/collision (V)** | **MRM transition (*m/z*)** |
| --- | --- | --- |
| Cer 18:1;O2/16:0 | 28/26 | 538.5 → 264.3 |
| Cer 18:1;O2/18:0 | 30/26 | 566.5 → 264.3 |
| Cer 18:1;O2/20:1 | 28/26 | 592.3 → 264.3 |
| Cer 18:1;O2/20:0 | 28/26 | 594.3 → 264.3 |
| Cer 18:1;O2/22:1 | 30/30 | 620.6 → 264.3 |
| Cer 18:1;O2/22:0 | 30/30 | 622.6 → 264.3 |
| Cer 18:1;O2/24:1 | 28/30 | 648.6 → 264.3 |
| Cer 18:1;O2/24:0 | 34/26 | 650.6 → 264.3 |
| HexCer 18:1;O2/16:0 | 26/38 | 700.5 → 264.3 |
| HexCer 18:1;O2/24:1 | 26/40 | 810.7 → 264.3 |
| HexCer 18:1;O2/24:0 | 26/40 | 812.8 → 264.3 |
| Hex2Cer 18:1;O2/16:0 | 30/44 | 862.6 → 264.3 |
| Hex2Cer 18:1;O2/24:1 | 32/50 | 972.7 → 264.3 |
| Hex2Cer 18:1;O2/24:0 | 36/50 | 974.7 → 264.3 |
| DihydroCer 18:0;O2/16:0 | 50/40 | 541.0 → 266.3 |
| DihydroCer 18:0;O2/24:0 | 50/40 | 652.8 → 266.3 |
| DihydroCer 18:0;O2/24:1 | 50/30 | 650.8 → 266.3 |
| Cer 18:1;O2/17:0, IS | 28/28 | 552.5 → 264.3 |
| SM 32:1;O2 | 58/26 | 675.6 → 184.1 |
| SM 34:2;O2 | 58/26 | 701.6 → 184.1 |
| SM 34:1;O2 | 58/26 | 703.6 → 184.1 |
| SM 36:2;O2 | 58/32 | 729.5 → 184.1 |
| SM 36:1;O2 | 58/32 | 731.5 → 184.1 |
| SM 38:2;O2 | 46/26 | 757.7 → 184.1 |
| SM 38:1;O2 | 46/26 | 759.7 → 184.1 |
| SM 40:2;O2 | 40/30 | 785.7 → 184.1 |
| SM 40:1;O2 | 40/30 | 787.7 → 184.1 |
| SM 42:2;O2 | 38/30 | 813.7 → 184.1 |
| SM 42:1;O2 | 36/30 | 815.7 → 184.1 |
| SM 35:1;O2 SI | 56/30 | 717.6 → 184.1 |

**Quantification of apolipoproteins –** Apolipoproteins (A-I, A-II, B100, C-I, C-II, C-II and E) were quantified in plasma and EV samples using trypsin proteolysis and the subsequent analysis of proteotypic peptides by LC-MS/MS. All solvents were LC-MS grade and purchased from Biosolve. Synthetic labeled and unlabeled proteotypic peptides were provided by Thermo Scientific Biopolymers (Darmstadt, Germany). Stock solutions (1 mM) were prepared in 50% acetonitrile containing 0.1% formic acid. A mixed solution of unlabeled peptides was constituted and serially diluted in water to obtain seven standard solutions ranging from 1 to 5,000 nM. Labeled peptides were used as ISs. A mixed solution of ISs (35 µM) was prepared and added to digestion buffer (ammonium bicarbonate, 50 mM) to a final concentration of 1.75 µM. Samples were prepared with the ProteinWorks™ eXpress kit (Waters, Milford, MA), according to the manufacturer’s instructions. Samples (40 µL) were incubated for 10 min at 80 °C in digestion buffer containing ISs (100 µL) and RapidGest detergent solution (7 mg/ml, 10 µL), reduced for 20 min at 60 °C with dithiothreitol (70 mM, 20 µL), alkylated for 30 min at room temperature in the dark with iodoacetamide (142 mM, 30 µL), and digested overnight at 37 °C with trypsin (7 mg/ml, 30 µL). Enzymatic digestion was stopped with 20% trifluoroacetic acid (TFA; 5 µL). After 15 min at 45 °C, the precipitate was removed by centrifugation (15 min; 10 °C; 10,000 g), and supernatants were cleaned on 30 mg Oasis HLB cartridges (Waters Corporation), which were conditioned (100% methanol; 1 mL), equilibrated (100% water; 1 mL), loaded (sample; 200 µL), washed (5% methanol; 1 mL), and eluted (80% methanol; 500 µL). The eluates were dried under nitrogen (45 °C) and reconstituted with 5% acetonitrile containing 0.1% formic acid (100 µL). Analyses were performed on a Xevo® TQD mass spectrometer with an ESI interface and an Acquity H-Class® UPLC™ device (Waters Corporation). Samples were injected (10 µL) onto an Acquity® BEH C18 column (2.1 × 100 mm, 1.7 µm, Waters Corporation) held at 60 °C. Compounds were separated with a linear gradient of mobile phase B (100% acetonitrile, 0.1% formic) in mobile phase A (5% acetonitrile, 0.1% formic acid) at a flow rate of 250 μL/min. Mobile phase B was kept constant for 0.5 min at 1%, linearly increased from 1% to 80% for 7 min, kept constant for 1.5 min, returned to the initial condition over 1 min, and kept constant for 2 min before the next injection. Proteolytic peptides and IS were detected by the mass spectrometer with the ESI interface operating in the positive ion mode (capillary voltage, 3 kV; desolvatation gas (N_2_) flow, 550 L/h; desolvatation gas temperature, 450 °C; source temperature, 120 °C). The multiple reaction mode (MRM) was applied as described in Supplemental Table S3. Chromatographic peak area ratios between peptides and their respective IS constituted the detector responses. Standard samples were used to plot calibration curves and a linear regression model (1/x weighting) was used for quantification.

**Supplemental Table S3.** MRM parameters used for apolipoprotein detection.

| **Apolipoproteins** | **Peptide sequence** | **Cone/collision (V)** | **MRM transition (*m/z*)** |
| --- | --- | --- | --- |
| ApoA-I | ATEHLSTLSEK | 25/15 | 406,2 → 573,2 |
| ApoA-I (SI) | ATEHLSTLSE-[^13^C_6_, ^15^N_2_]K | 25/15 | 408,9 → 577,2 |
| ApoA-II | SPELQAEAK | 30/15 | 486,8 → 659,3 |
| ApoA-II (SI) | SPELQAEA-[^13^C_6_, ^15^N_2_]K | 30/15 | 490,3 → 667,3 |
| ApoB100 | ATGVLYDYVNK | 34/23 | 622,4 → 915,6 |
| ApoB100 (SI) | ATGVLYDYVN-[^13^C_6_, ^15^N_2_]K | 34/23 | 626,4 → 923,6 |
| ApoC-I | TPDVSSALDK | 40/23 | 517,4 → 466,8 |
| ApoC-I (SI) | TPDVSSALD-[^13^C_6_, ^15^N_2_]K | 40/23 | 621,4 → 470,8 |
| ApoC-II | TAAQNLYEK | 35/20 | 519,7 → 865,7 |
| ApoC-II (SI) | TAAQNLYE-[^13^C_6_, ^15^N_2_]K | 35/20 | 523,7 → 873,7 |
| ApoC-III | GWVTDGFSSLK | 30/22 | 599,2 → 854,8 |
| ApoC-III (SI) | GWVTDGFSSL-[^13^C_6_, ^15^N_2_]K | 30/22 | 603,2 → 862,8 |
| ApoE | LGPLVEQGR | 25/30 | 484,8 → 588,3 |
| ApoE (SI) | LGPLVEQG-[^13^C_6_, ^15^N_4_]R | 25/30 | 489,8 → 598,3 |
