# Supplementary material for: Monitoring concentration and lipid signature of plasma extracellular vesicles from HR+ metastatic breast cancer patients under CDK4/6 inhibitors treatment
Source: J Extracell Biol. 2024 Dec 17;3(12):e70013. doi: 10.1002/jex2.70013 (PMC11650302; doi:10.1002/jex2.70013)
Supplement: Supplementary file 2 — Supporting Information [file JEX2-3-e70013-s002.docx]

**Extended data Figure 1. Individual graphs of HR^+^ MBC patients for the follow up of the vesiclemia, associated with the therapeutic lines they received.**

**Extended data Figure 2. Plasma EV concentration at screening time in different groups of patients, associated with treatments. A.** Vesiclemia measured by ILM in healthy donors (n=45), hormone receptor-positive (HR^+^) patients who received CDK4/6 inhibitor treatment as first line (n=51), or not (n=17), HER2^+^ patients (n=15) and triple negative breast cancer (TNBC) patients (n=8). **B.** Cryo-Transmission Electron Microscopy (Cryo-TEM) images of plasma EVs from a MBC patient (n=55 measured EVs, average size: 116 ± 63nm) (bar scale = 100nm). This is representative of n=3 individual samples. **C.** Vesiclemia at screening time in HR^+^ MBC patients, initially metastatic (n=24, average vesiclemia: 9.5 ± 8.2x10^9^) or not (n=46, average vesiclemia: 8.4 ± 6.8x10^9^). T-test and ANOVA: ns, not significant.

**Extended data Figure 3. Targeted lipidomic analysis of EVs plasma from healthy subjects and MBC patients. A.** Principal component analysis represents quantitative analysis of triacylglycerols (n=44) by mass spectrometry from EV samples enriched after SEC from n=4 healthy subjects, n=4 sensitive, n=4 intermediate and n=3. **B.** HeatMap represents the intensity of each triacylglycerol (n=44) concentration per individual (n=15). **C.** Proportion of each one of the 27 sphingolipids detected in EV samples at screening time per groups (n=6 sensitive patients, n=6 intermediate patients and n=6 resistant patients). The proportion of each lipid was calculated as the ratio of its concentration to the total concentration of sphingolipids. Statistical analyses were performed using the T-test and two-way ANOVA test.

**Extended data Figure 4. Lipid analysis of plasma EVs and plasma from healthy subjects and MBC patients 2 months after treatment initiation. A.** Proportion of each ceramide (n=8) quantitatively analyzed in n=11 plasma samples from MBC patients. **B.** Proportion of each sphingomyelin (n=11) quantitatively analyzed in n=11 plasma samples from MBC patients. **C.** The graph shows the correlation between the total concentrations of 2 classes of sphingolipids (ceramides, n=8 and sphingomyelins, n=11) in n=27 MBC patients’ plasma EVs samples and, **D.** directly in plasma of n=11 MBC patients. **E.** Proportion of each one of the 5 ceramides (1) measured directly in plasma by group (n=3 healthy donors, n=3 sensitive patients, n=5 intermediate patients and n=3 resistant patients). Cer (1) denotes ceramides containing one double bond. The proportion of each lipid was calculated by first averaging the concentrations for each patient within their respective groups. Then, the average concentration for each patient group was divided by the overall average concentration of all Cer (1). Thus, each point represents a lipid species **F.** Proportion of each one of the 3 ceramides (2) measured directly in plasma by group (n=3 healthy donors, n=3 sensitive patients, n=5 intermediate patients and n=3 resistant patients). Cer (2) denotes ceramides containing two double bonds. The proportion of each lipid was calculated by first averaging the concentrations for each patient within their respective groups. Then, the average concentration for each patient group was divided by the overall average concentration of all Cer (2). Thus, each point represents a lipid species. **G.** Proportion of each one of the 6 sphingomyelins (1) measured directly in plasma by group (n=3 healthy donors, n=3 sensitive patients, n=5 intermediate patients and n=3 resistant patients). SM (1) denotes sphingomyelins containing one double bond. The proportion of each lipid was calculated by first averaging the concentrations for each patient within their respective groups. Then, the average concentration for each patient group was divided by the overall average concentration of all SM (1). Thus, each point represents a lipid species. **H.** Proportion of each one of the 5 sphingomyelins (2) measured directly in plasma by group (n=3 healthy donors, n=3 sensitive patients, n=5 intermediate patients and n=3 resistant patients). SM (2) denotes sphingomyelins containing two double bonds. The proportion of each lipid was calculated by first averaging the concentrations for each patient within their respective groups. Then, the average concentration for each patient group was divided by the overall average concentration of all SM (2). Thus, each point represents a lipid species. **I.** Concentration (nmol/L) of the ceramide 18:1;O2/24:0 in each group of patients (n=10 sensitive patients, n=10 intermediate patients and n=7 resistant patients) at visit 1 in EV samples. **J.** Concentration (nmol/L) of the sphingomyelin 40:1;O2 in each group of patients (n=10 sensitive patients, n=10 intermediate patients and n=7 resistant patients) at visit 1 in EV samples. **K.** Concentration (nmol/L) of the ceramide 18:1;O2/24:0 in healthy subjects (n=3) and in each group of patients (n=3 sensitive patients, n=5 intermediate patients and n=3 resistant patients) at visit 1 directly in plasma. **L.** Concentration (nmol/L) of the sphingomyelin 40:1;O2 in healthy subjects (n=3) and in each group of patients (n=3 sensitive patients, n=5 intermediate patients and n=3 resistant patients) at visit 1 directly in plasma All panels are representative of at least three independent experiments unless otherwise stated, Student test and ANOVA, *p<0.05, **p<0.01, ****p<0.0001.

**Extended data table I.** Identification of lipid categories and classes.
