# Supplementary material for: Monitoring concentration and lipid signature of plasma extracellular vesicles from HR+ metastatic breast cancer patients under CDK4/6 inhibitors treatment
Source: J Extracell Biol. 2024 Dec 17;3(12):e70013. doi: 10.1002/jex2.70013 (PMC11650302; doi:10.1002/jex2.70013)
Supplement: Supplementary file 5 — Supporting Information [file JEX2-3-e70013-s003.pptx]

## Slide 1
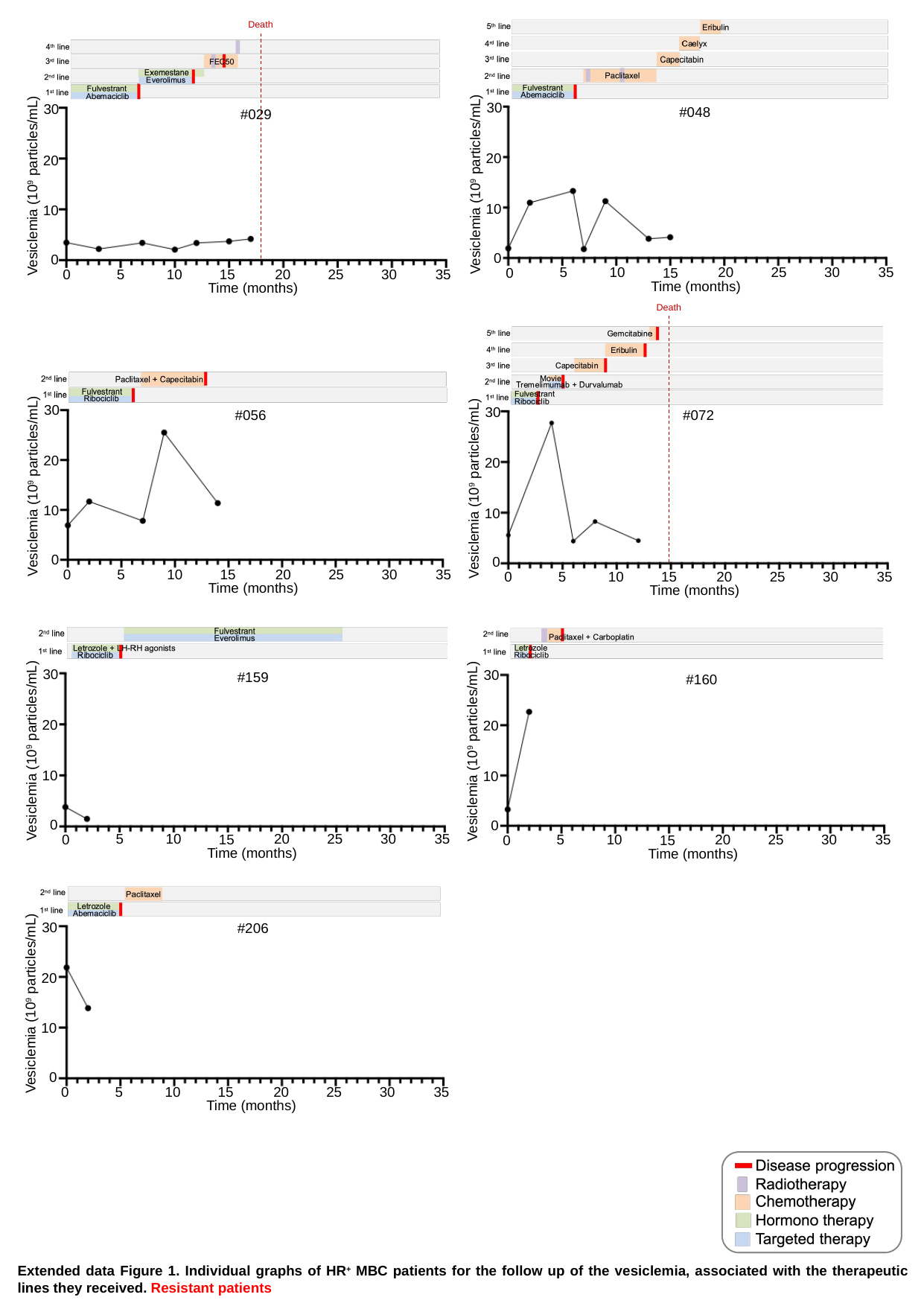

Death
30
20
Vesiclemia (109 particles/mL)
10
0
5
10
20
25
30
35
15
0
Time (months)
#029
30
20
Vesiclemia (109 particles/mL)
10
0
5
10
20
25
30
35
15
0
Time (months)
#048
Death
30
20
Vesiclemia (109 particles/mL)
10
0
5
10
20
25
30
35
15
0
Time (months)
#072
30
20
Vesiclemia (109 particles/mL)
10
0
5
10
20
25
30
35
15
0
Time (months)
#056
30
20
Vesiclemia (109 particles/mL)
10
0
5
10
20
25
30
35
15
0
Time (months)
#159
30
20
Vesiclemia (109 particles/mL)
10
0
5
10
20
25
30
35
15
0
Time (months)
#160
30
20
Vesiclemia (109 particles/mL)
10
0
5
10
20
25
30
35
15
0
Time (months)
#206
Extended data Figure 1. Individual graphs of HR+ MBC patients for the follow up of the vesiclemia, associated with the therapeutic lines they received. Resistant patients

## Slide 2
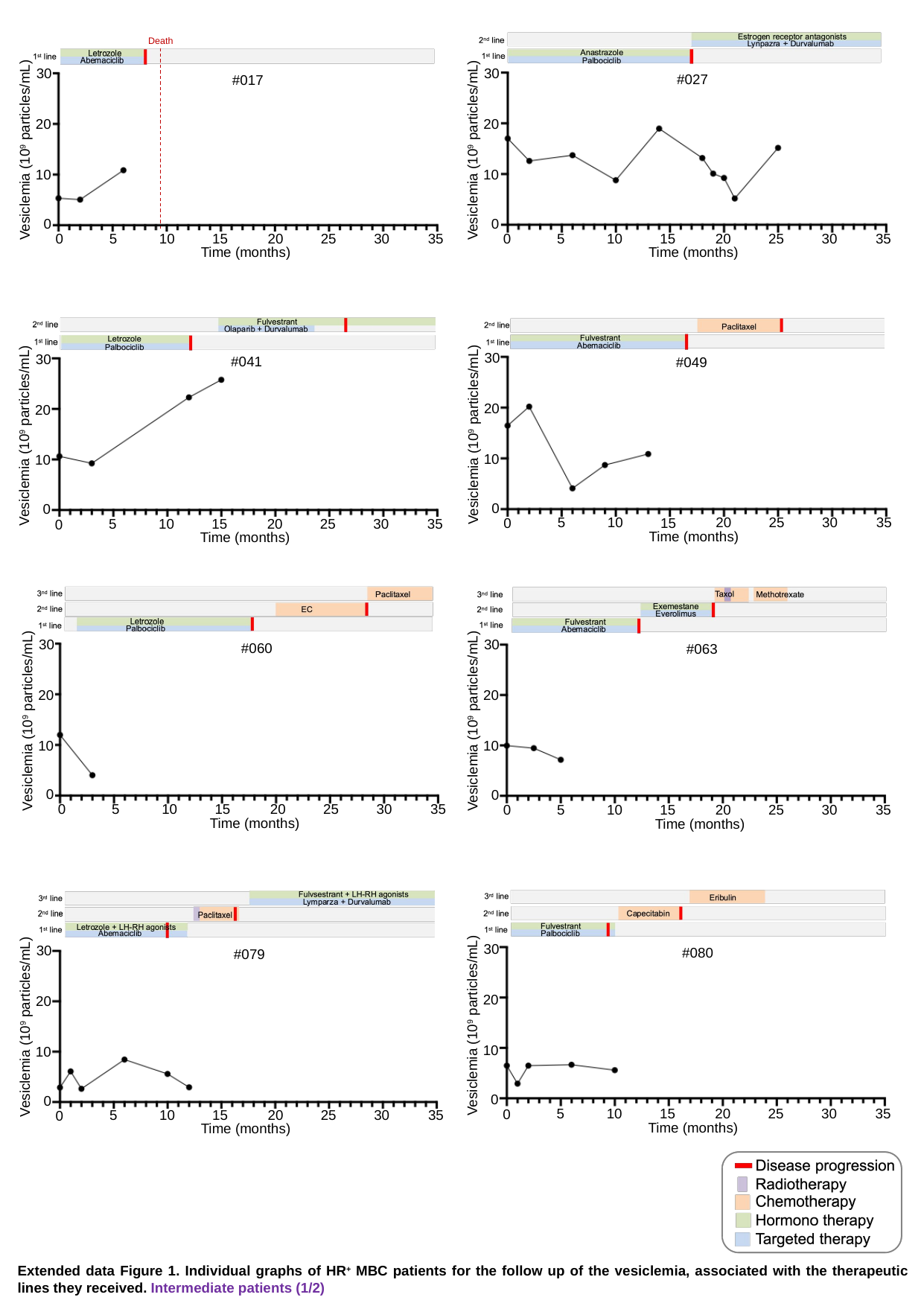

30
20
Vesiclemia (109 particles/mL)
10
0
5
10
20
25
30
35
15
0
Time (months)
#027
Death
30
20
Vesiclemia (109 particles/mL)
10
0
5
10
20
25
30
35
15
0
Time (months)
#017
30
20
Vesiclemia (109 particles/mL)
10
0
5
10
20
25
30
35
15
0
Time (months)
#041
30
20
Vesiclemia (109 particles/mL)
10
0
5
10
20
25
30
35
15
0
Time (months)
#049
30
20
Vesiclemia (109 particles/mL)
10
0
5
10
20
25
30
35
15
0
Time (months)
#060
30
20
Vesiclemia (109 particles/mL)
10
0
5
10
20
25
30
35
15
0
Time (months)
#063
30
20
Vesiclemia (109 particles/mL)
10
0
5
10
20
25
30
35
15
0
Time (months)
#080
30
20
Vesiclemia (109 particles/mL)
10
0
5
10
20
25
30
35
15
0
Time (months)
#079
Extended data Figure 1. Individual graphs of HR+ MBC patients for the follow up of the vesiclemia, associated with the therapeutic lines they received. Intermediate patients (1/2)

## Slide 3
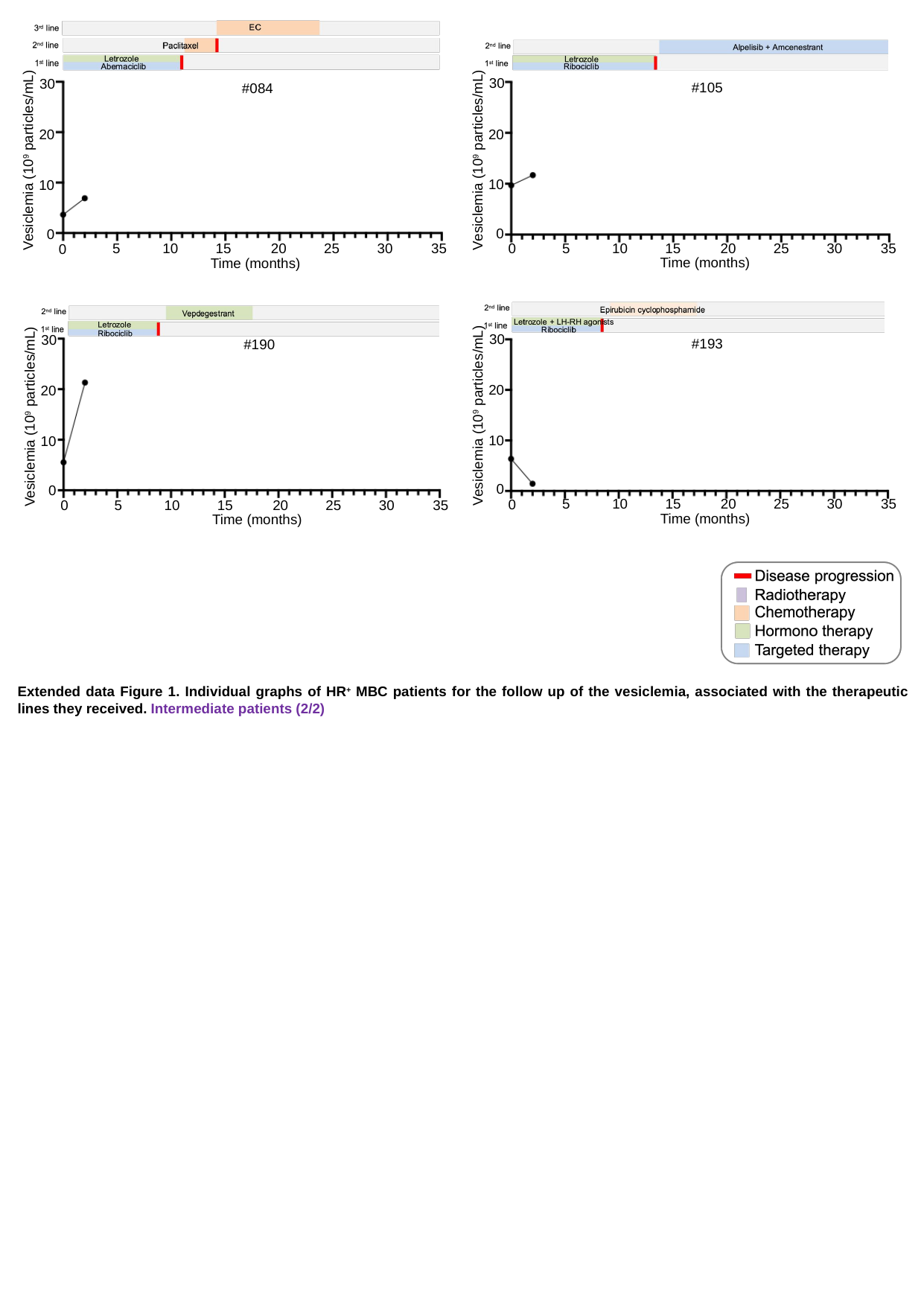

30
20
Vesiclemia (109 particles/mL)
10
0
5
10
20
25
30
35
15
0
Time (months)
#084
30
20
Vesiclemia (109 particles/mL)
10
0
5
10
20
25
30
35
15
0
Time (months)
#105
30
20
Vesiclemia (109 particles/mL)
10
0
5
10
20
25
30
35
15
0
Time (months)
#193
30
20
Vesiclemia (109 particles/mL)
10
0
5
10
20
25
30
35
15
0
Time (months)
#190
Extended data Figure 1. Individual graphs of HR+ MBC patients for the follow up of the vesiclemia, associated with the therapeutic lines they received. Intermediate patients (2/2)

## Slide 4
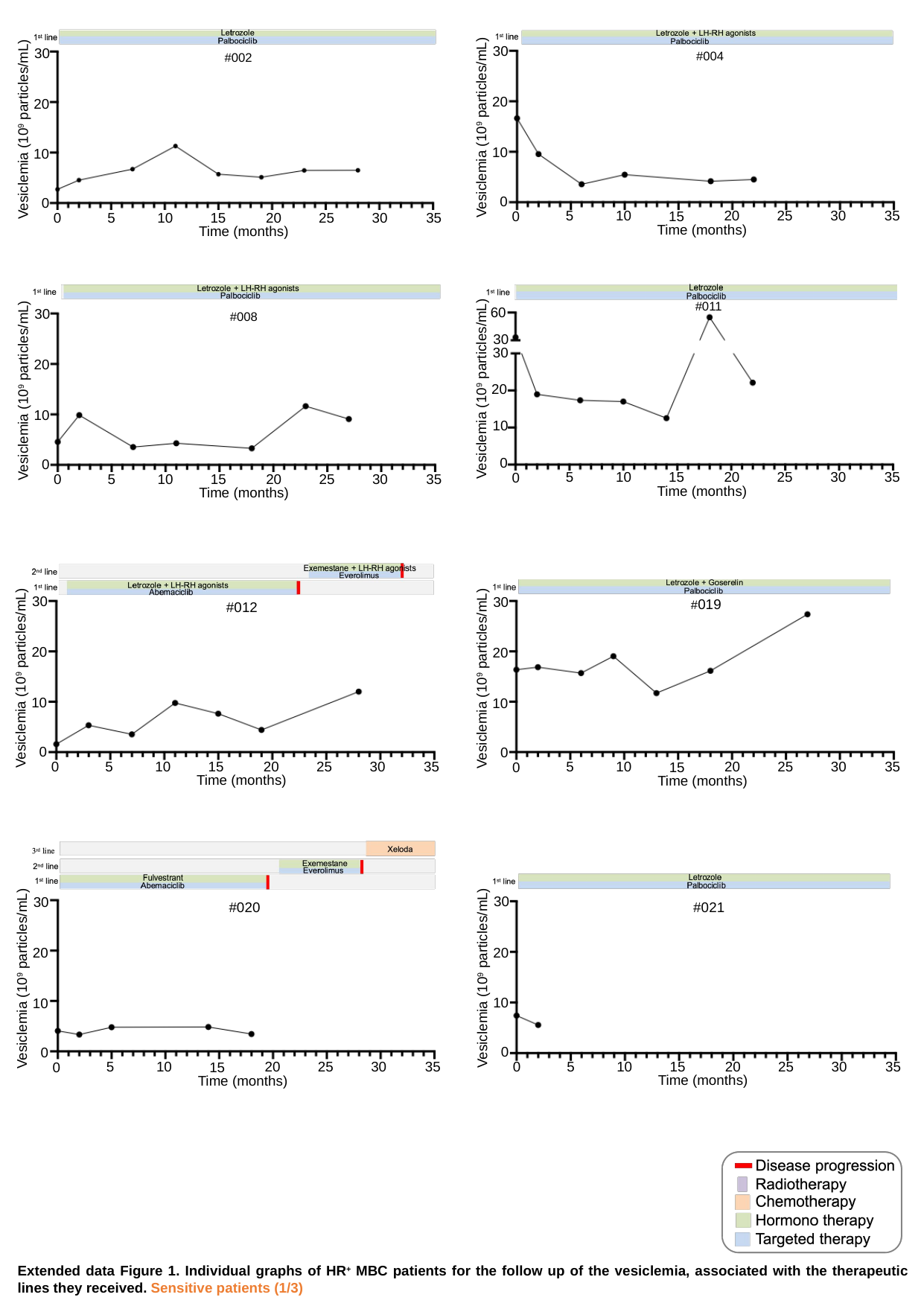

30
20
Vesiclemia (109 particles/mL)
10
0
5
10
20
25
30
35
15
0
Time (months)
#002
30
20
Vesiclemia (109 particles/mL)
10
0
5
10
20
25
30
35
15
0
Time (months)
#004
30
20
Vesiclemia (109 particles/mL)
10
0
5
10
20
25
30
35
15
0
Time (months)
#008
30
20
Vesiclemia (109 particles/mL)
10
0
5
10
20
25
30
35
15
0
Time (months)
#011
60
30
30
20
Vesiclemia (109 particles/mL)
10
0
5
10
20
25
30
35
15
0
Time (months)
#012
30
20
Vesiclemia (109 particles/mL)
10
0
5
10
20
25
30
35
15
0
Time (months)
#019
30
20
Vesiclemia (109 particles/mL)
10
0
5
10
20
25
30
35
15
0
Time (months)
#020
30
20
Vesiclemia (109 particles/mL)
10
0
5
10
20
25
30
35
15
0
Time (months)
#021
Extended data Figure 1. Individual graphs of HR+ MBC patients for the follow up of the vesiclemia, associated with the therapeutic lines they received. Sensitive patients (1/3)

## Slide 5
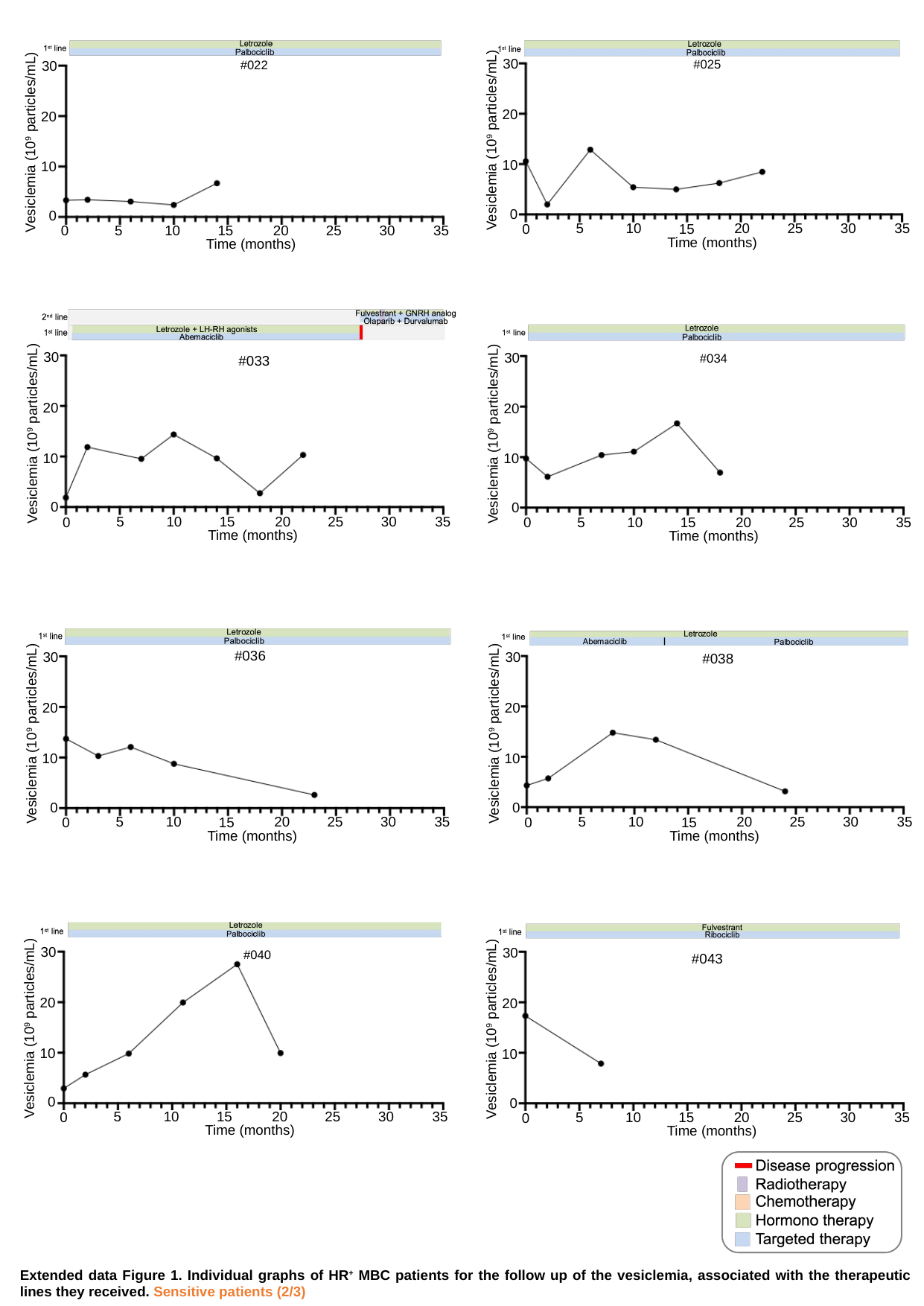

30
20
Vesiclemia (109 particles/mL)
10
0
5
10
20
25
30
35
15
0
Time (months)
#022
30
20
Vesiclemia (109 particles/mL)
10
0
5
10
20
25
30
35
15
0
Time (months)
#025
30
20
Vesiclemia (109 particles/mL)
10
0
5
10
20
25
30
35
15
0
Time (months)
#033
30
20
Vesiclemia (109 particles/mL)
10
0
5
10
20
25
30
35
15
0
Time (months)
#034
30
20
Vesiclemia (109 particles/mL)
10
0
5
10
20
25
30
35
15
0
Time (months)
#036
30
20
Vesiclemia (109 particles/mL)
10
0
5
10
20
25
30
35
15
0
Time (months)
#038
30
20
Vesiclemia (109 particles/mL)
10
0
5
10
20
25
30
35
15
0
Time (months)
#040
30
20
Vesiclemia (109 particles/mL)
10
0
5
10
20
25
30
35
15
0
Time (months)
#043
Extended data Figure 1. Individual graphs of HR+ MBC patients for the follow up of the vesiclemia, associated with the therapeutic lines they received. Sensitive patients (2/3)

## Slide 6
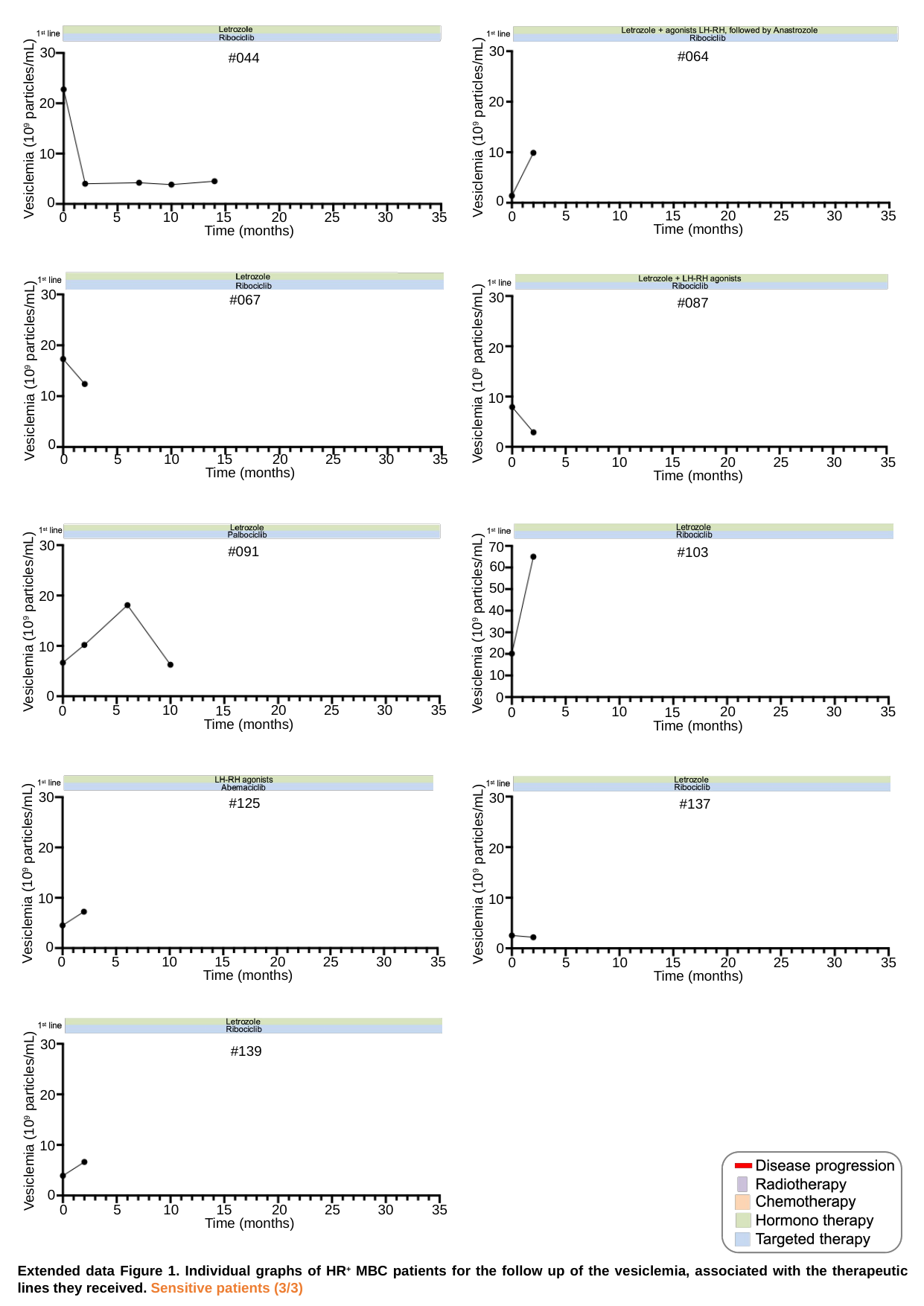

30
20
Vesiclemia (109 particles/mL)
10
0
5
10
20
25
30
35
15
0
Time (months)
#044
30
20
Vesiclemia (109 particles/mL)
10
0
5
10
20
25
30
35
15
0
Time (months)
#064
30
20
Vesiclemia (109 particles/mL)
10
0
5
10
20
25
30
35
15
0
Time (months)
#067
30
20
Vesiclemia (109 particles/mL)
10
0
5
10
20
25
30
35
15
0
Time (months)
#087
70
Vesiclemia (109 particles/mL)
20
10
0
5
10
20
25
30
35
15
0
Time (months)
#103
60
50
40
30
30
20
Vesiclemia (109 particles/mL)
10
0
5
10
20
25
30
35
15
0
Time (months)
#091
30
20
Vesiclemia (109 particles/mL)
10
0
5
10
20
25
30
35
15
0
Time (months)
#137
30
20
Vesiclemia (109 particles/mL)
10
0
5
10
20
25
30
35
15
0
Time (months)
#125
30
20
Vesiclemia (109 particles/mL)
10
0
5
10
20
25
30
35
15
0
Time (months)
#139
Extended data Figure 1. Individual graphs of HR+ MBC patients for the follow up of the vesiclemia, associated with the therapeutic lines they received. Sensitive patients (3/3)

## Slide 7
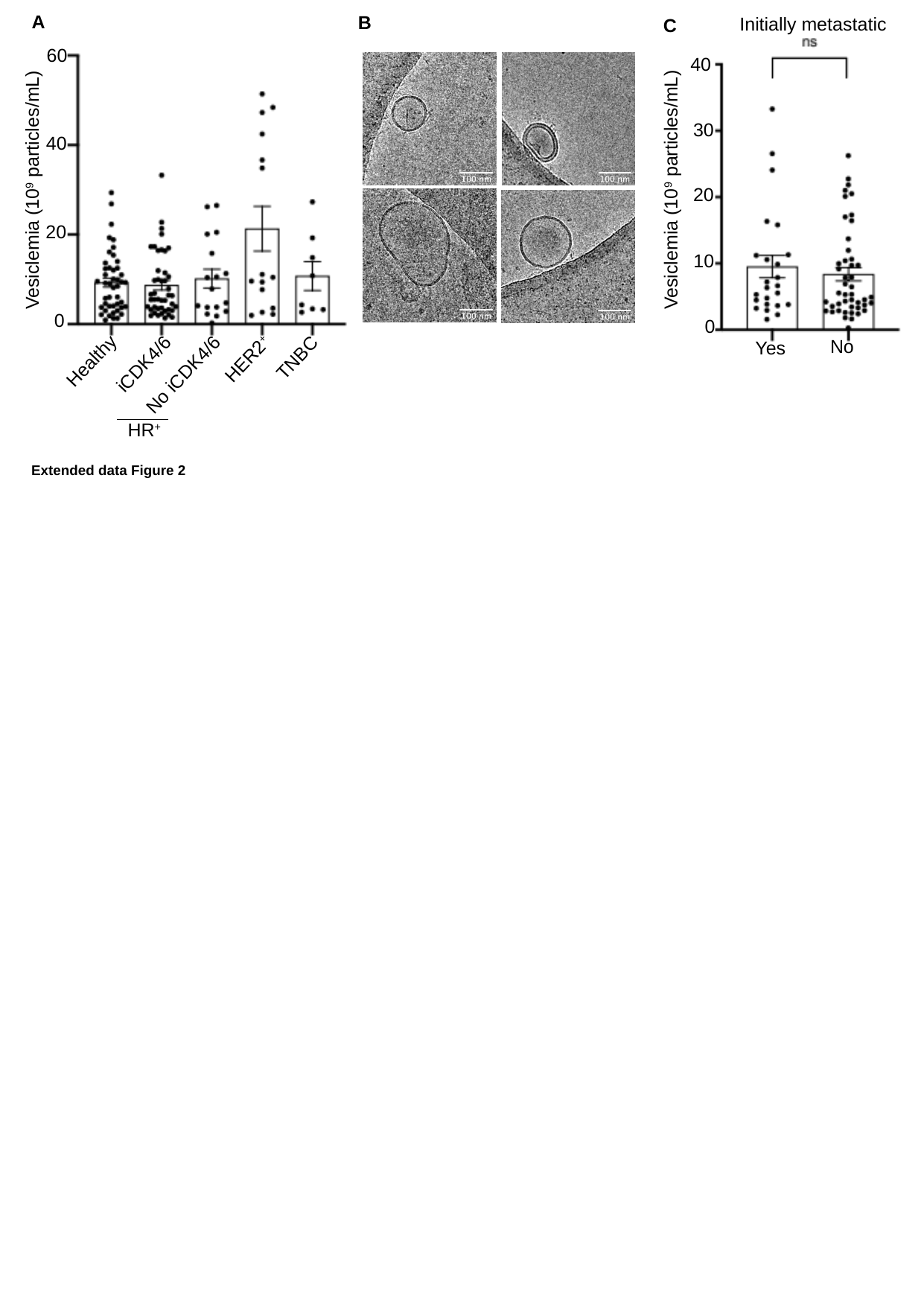

60
40
Vesiclemia (109 particles/mL)
20
0
HR+
TNBC
HER2+
Healthy
A
iCDK4/6
No iCDK4/6
B
Initially metastatic
40
30
20
0
No
Yes
Vesiclemia (109 particles/mL)
10
C
Extended data Figure 2

## Slide 8
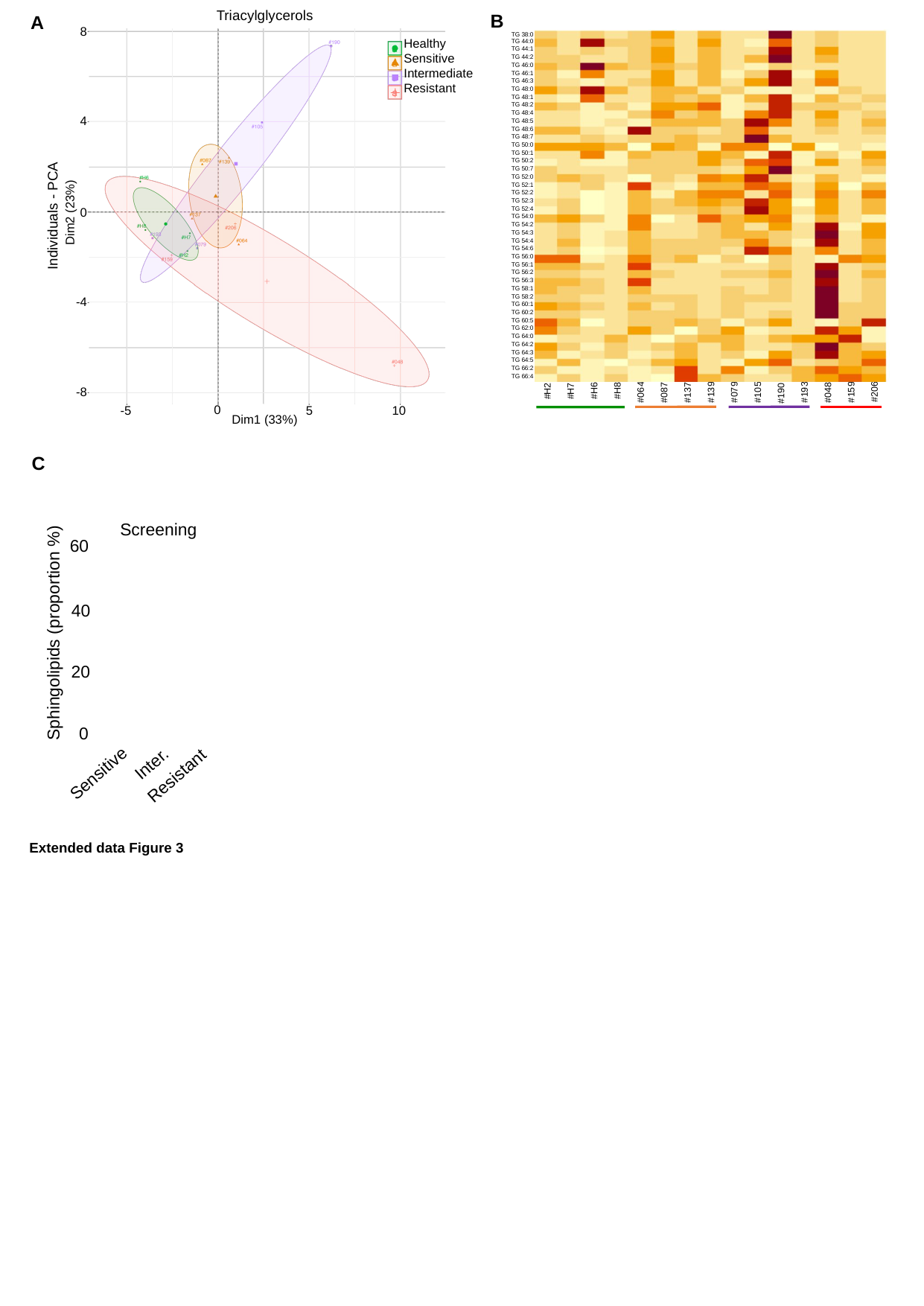

Triacylglycerols
A
Healthy
Sensitive
Intermediate
Resistant
Individuals - PCA
8
4
0
Dim2 (23%)
-4
-8
0
10
-5
5
Dim1 (33%)
B
TG 38:0
TG 44:0
TG 44:1
TG 44:2
TG 46:0
TG 46:1
TG 46:3
TG 48:0
TG 48:1
TG 48:2
TG 48:4
TG 48:5
TG 48:6
TG 48:7
TG 50:0
TG 50:1
TG 50:2
TG 50:7
TG 52:0
TG 52:1
TG 52:2
TG 52:3
TG 52:4
TG 54:0
TG 54:2
TG 54:3
TG 54:4
TG 54:6
TG 56:0
TG 56:1
TG 56:2
TG 56:3
TG 58:1
TG 58:2
TG 60:1
TG 60:2
TG 60:5
TG 62:0
TG 64:0
TG 64:2
TG 64:3
TG 64:5
TG 66:2
TG 66:4
#H2
#H7
#H6
#H8
#206
#064
#087
#137
#139
#079
#105
#193
#048
#159
#190
C
60
Sphingolipids (proportion %)
20
0
Inter.
Sensitive
Resistant
40
Screening
Extended data Figure 3

## Slide 9
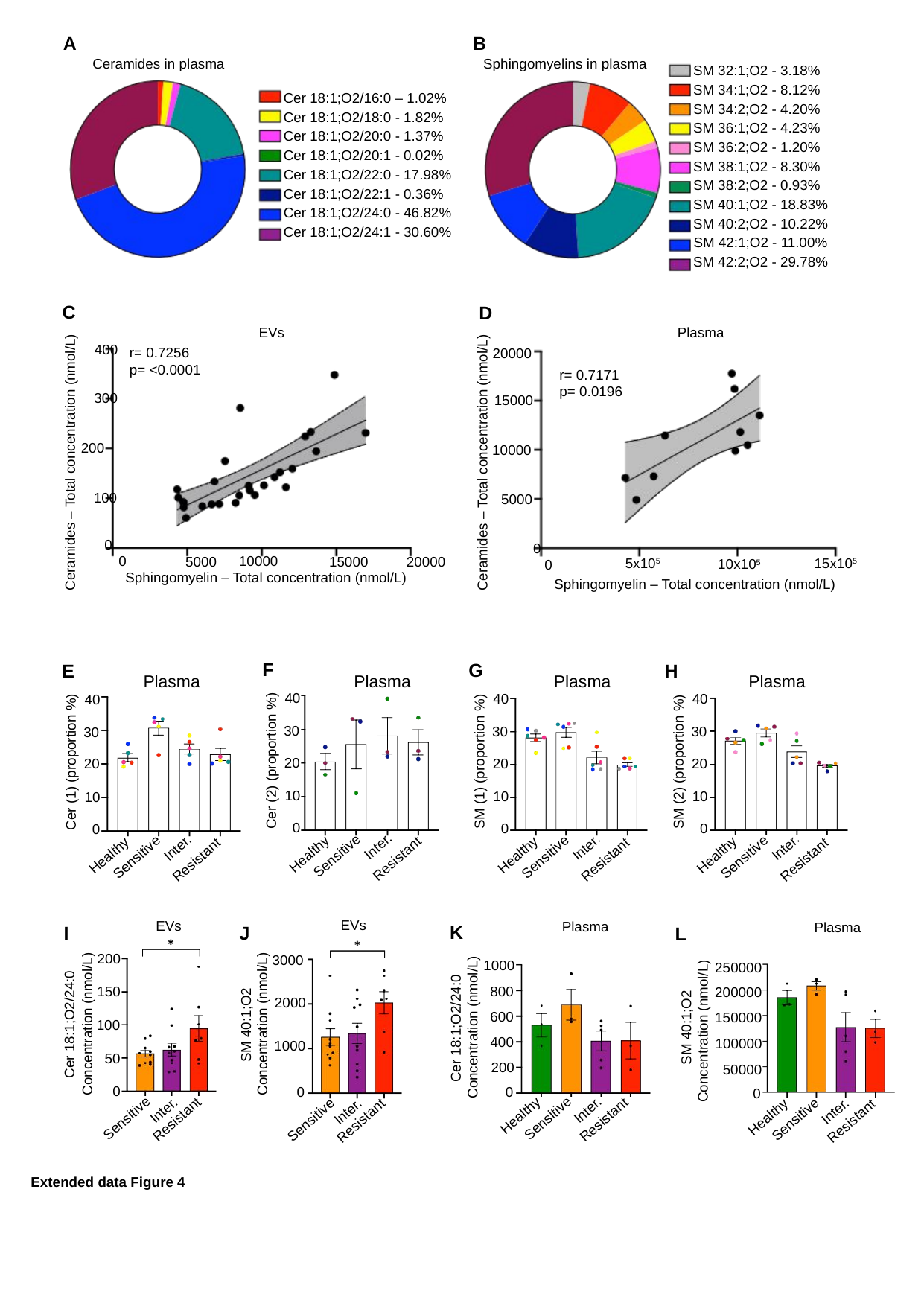

A
Ceramides in plasma
Cer 18:1;O2/16:0 – 1.02%
Cer 18:1;O2/18:0 - 1.82%
Cer 18:1;O2/20:0 - 1.37%
Cer 18:1;O2/20:1 - 0.02%
Cer 18:1;O2/22:0 - 17.98%
Cer 18:1;O2/22:1 - 0.36%
Cer 18:1;O2/24:0 - 46.82%
Cer 18:1;O2/24:1 - 30.60%
B
Sphingomyelins in plasma
SM 32:1;O2 - 3.18%
SM 34:1;O2 - 8.12%
SM 34:2;O2 - 4.20%
SM 36:1;O2 - 4.23%
SM 36:2;O2 - 1.20%
SM 38:1;O2 - 8.30%
SM 38:2;O2 - 0.93%
SM 40:1;O2 - 18.83%
SM 40:2;O2 - 10.22%
SM 42:1;O2 - 11.00%
SM 42:2;O2 - 29.78%
C
400
300
200
Ceramides – Total concentration (nmol/L)
100
0
0
10000
5000
15000
Sphingomyelin – Total concentration (nmol/L)
r= 0.7256
p= <0.0001
20000
D
20000
15000
10000
Ceramides – Total concentration (nmol/L)
5000
0
0
Sphingomyelin – Total concentration (nmol/L)
r= 0.7171
p= 0.0196
Plasma
5x10^5^
15x105
10x105
EVs
F
40
Cer (2) (proportion %)
20
0
Inter.
Sensitive
Resistant
30
10
Healthy
G
Plasma
40
SM (1) (proportion %)
20
0
Inter.
Sensitive
Resistant
30
10
Healthy
H
40
SM (2) (proportion %)
20
0
Inter.
Sensitive
Resistant
30
10
Healthy
E
40
Cer (1) (proportion %)
20
0
Inter.
Sensitive
Resistant
30
10
Healthy
Plasma
Plasma
Plasma
EVs
EVs
Plasma
1000
Concentration (nmol/L)
400
0
Inter.
Healthy
Sensitive
Resistant
800
200
600
K
Cer 18:1;O2/24:0
Plasma
250000
Concentration (nmol/L)
100000
0
Inter.
Healthy
Sensitive
Resistant
200000
50000
150000
L
SM 40:1;O2
J
3000
Concentration (nmol/L)
0
Inter.
Sensitive
Resistant
2000
SM 40:1;O2
1000
I
200
Concentration (nmol/L)
0
Inter.
Sensitive
Resistant
150
50
Cer 18:1;O2/24:0
100
Extended data Figure 4

## Slide 10
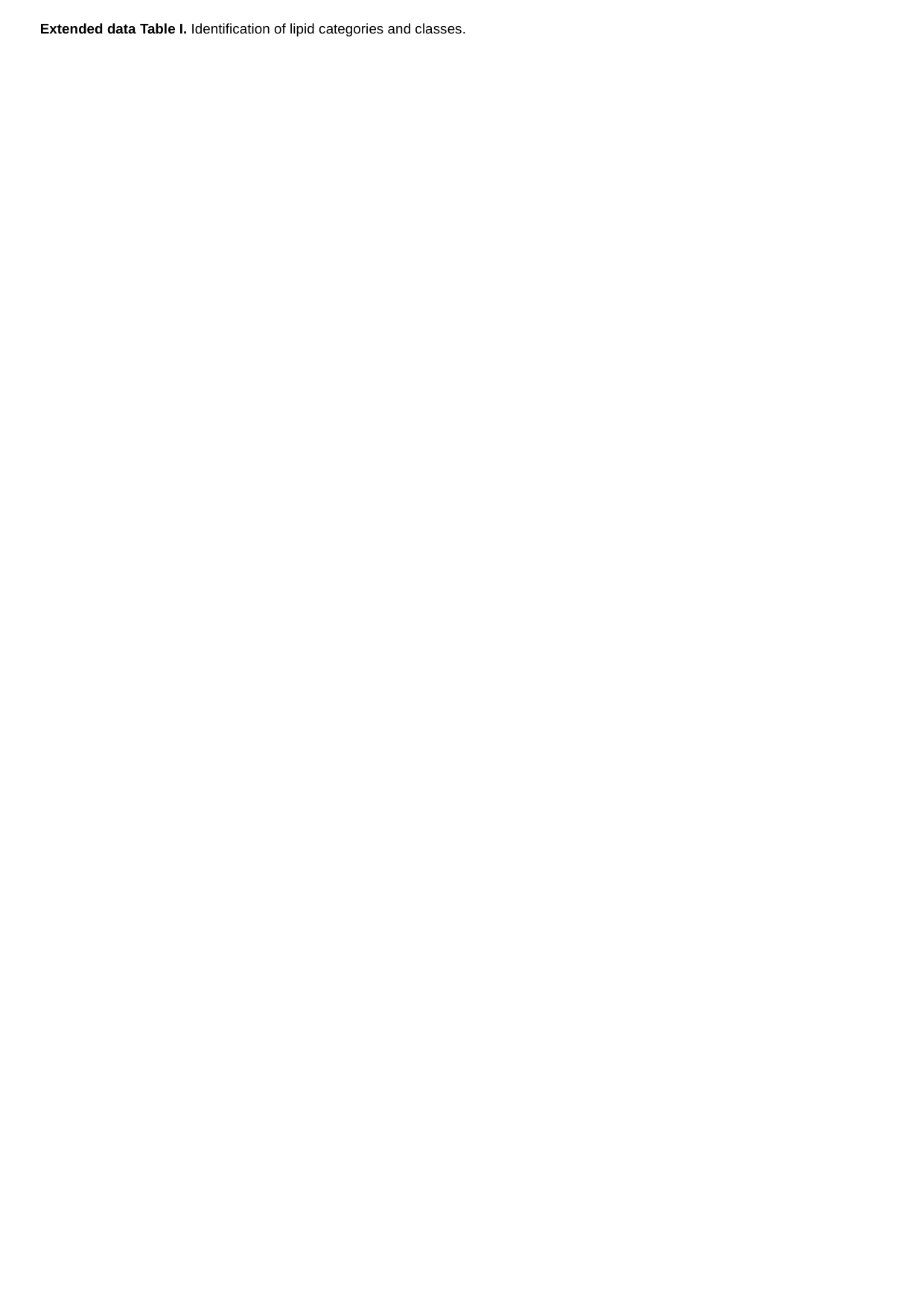

Extended data Table I. Identification of lipid categories and classes.
